# The Effects of Depth Cues and Vestibular Translation Signals on the Rotation Tolerance of Heading Tuning in Macaque Area MSTd

**DOI:** 10.1523/ENEURO.0259-20.2020

**Published:** 2020-11-16

**Authors:** Adam D. Danz, Dora E. Angelaki, Gregory C. DeAngelis

**Affiliations:** 1 Department of Brain and Cognitive Sciences, Center for Visual Science, University of Rochester, Rochester, NY 14627; 2 Center for Neural Science, New York University, New York, NY 10003

**Keywords:** extrastriate cortex, motion, navigation, optic flow, self-motion, smooth pursuit

## Abstract

When the eyes rotate during translational self-motion, the focus of expansion (FOE) in optic flow no longer indicates heading, yet heading judgements are largely unbiased. Much emphasis has been placed on the role of extraretinal signals in compensating for the visual consequences of eye rotation. However, recent studies also support a purely visual mechanism of rotation compensation in heading-selective neurons. Computational theories support a visual compensatory strategy but require different visual depth cues. We examined the rotation tolerance of heading tuning in macaque area MSTd using two different virtual environments, a frontoparallel (2D) wall and a 3D cloud of random dots. Both environments contained rotational optic flow cues (i.e., dynamic perspective), but only the 3D cloud stimulus contained local motion parallax cues, which are required by some models. The 3D cloud environment did not enhance the rotation tolerance of heading tuning for individual MSTd neurons, nor the accuracy of heading estimates decoded from population activity, suggesting a key role for dynamic perspective cues. We also added vestibular translation signals to optic flow, to test whether rotation tolerance is enhanced by non-visual cues to heading. We found no benefit of vestibular signals overall, but a modest effect for some neurons with significant vestibular heading tuning. We also find that neurons with more rotation tolerant heading tuning typically are less selective to pure visual rotation cues. Together, our findings help to clarify the types of information that are used to construct heading representations that are tolerant to eye rotations.

## Significance Statement

To estimate one’s direction of translation (or heading) from optic flow, it is necessary for the brain to compensate for the effects of eye rotations on the optic flow field. We examined how visual depth cues and vestibular translation signals contribute to the rotation tolerance of heading tuning in macaque area MSTd. Unlike the prediction of some computational models, we find that motion parallax cues in a 3D environment have little effect on rotation tolerance of MSTd neurons. We also find that vestibular translation signals do not substantially enhance tolerance to rotation. Our findings support a dominant role for visual rotation (i.e., dynamic perspective) cues in constructing a rotation-tolerant representation of heading in MSTd.

## Introduction

**Figure 1.**
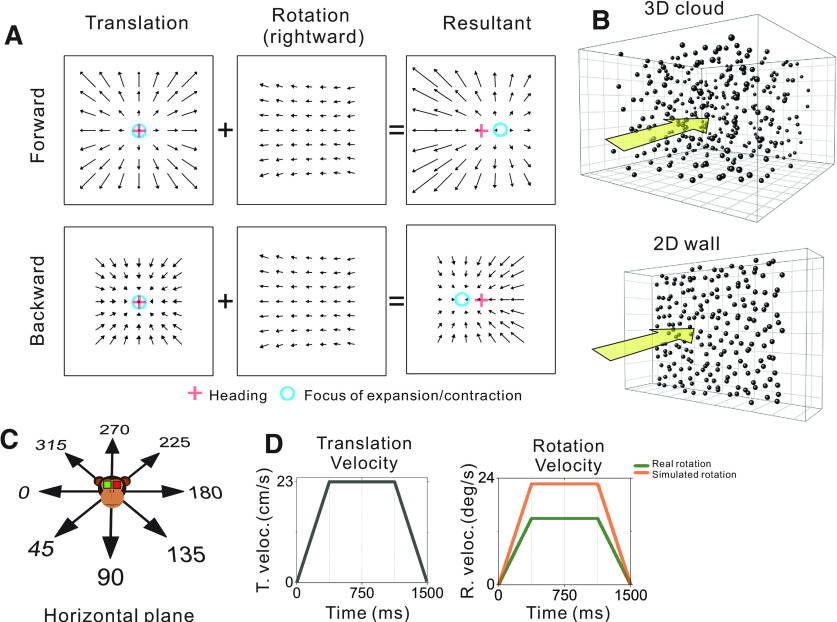
Schematic illustration of optic flow and experimental stimulus manipulations. ***A***, Optic flow patterns during self-motion shown under planar image projection. Pure translation (left) produces a radial expansion (upper) or contraction (lower) flow field for forward and backward headings, respectively. When a flow field produced by horizontal eye rotation (middle) is added, the FOE shifts in the direction of eye rotation for forward headings and the FOC shifts in the direction opposite to eye rotation during backward headings (right). ***B***, The virtual environment was either a 3D cloud of dots (top) or a 2D frontoparallel plane (bottom). ***C***, Real and simulated translation was presented in eight equally-spaced directions within the horizontal plane. ***D***, The velocity profiles for translation (left) and rotation (right) were constant during the middle 750 ms, which defined the analysis window.

Navigation through the environment produces an image velocity pattern on the retina, known as optic flow (Gibson, 1950), that is determined by translation and rotation of the eye relative to the world. In the absence of eye rotation and independent movement of objects in the scene, the direction of instantaneous translation, or heading, is related to the pattern of optic flow, with forward and backward translations indicated by a focus of expansion (FOE) or focus of contraction (FOC), respectively (Fig. 1*A*, left). Importantly, eye rotation distorts this radial pattern of optic flow such that the FOE and FOC no longer indicate heading (Fig. 1*A*, right); nevertheless, humans can estimate heading from optic flow quite accurately during eye rotations (Warren and Hannon, 1988; Royden et al., 1992). These observations motivated research on how the visual system discounts the rotational component of optic flow to estimate heading.

One strategy that has received considerable attention in both psychophysics (Royden et al., 1992, 1994; Crowell et al., 1998) and electrophysiology (Bradley et al., 1996; Page and Duffy, 1999; Shenoy et al., 1999; Sunkara et al., 2015) involves the contribution of extraretinal signals to constructing a rotation-tolerant representation of heading. It has been suggested that efference copies of motor commands or proprioceptive signals can be used to discount the rotational component of optic flow. To discount the net rotation of the eye relative to the world, this strategy would generally require integration of signals related to eye-in-head, head-on-body, and body-in-world rotations, potentially compounding the noise associated with each signal (Crowell et al., 1998).

Alternatively, the visual system could theoretically estimate eye-in-world rotation directly from optic flow. Local motion parallax cues created by pairs of neighboring objects at different depths can distinguish translational and rotational flow fields (Longuet-Higgins and Prazdny, 1980; Rieger and Lawton, 1985; Royden, 1997). Additionally, eye rotation causes perspective distortions of the flow field, also known as dynamic perspective cues, that can also be used to identify eye-in-world rotation (Koenderink and van Doorn, 1976; Grigo and Lappe, 1999; Kim et al., 2015). For example, eye rotation about the vertical axis results in leftward or rightward global motion on the spherical retina. However, when projected onto a planar image surface, the same eye rotation generates a component of vertical shearing motion that distinguishes eye rotation from eye translation (Kim et al., 2015). When the eye tracks a fixation point rightward across a frontoparallel background of dots, the right side of the background stimulus (under planar projection) will vertically contract while the left side will vertically expand (Kim et al., 2015, their Movie 3). These time-varying perspective distortions in the planar image projection provide information about the velocity of eye rotation. Together, motion parallax and dynamic perspective cues enable visual strategies for achieving rotation-tolerant heading perception and are supported by some psychophysical studies (Grigo and Lappe, 1999; Li and Warren, 2000, 2002; Crowell and Andersen, 2001).

While early electrophysiological studies supported extraretinal mechanisms of rotation compensation (for review, see Britten, 2008), some of these studies (Bradley et al., 1996; Shenoy et al., 1999) incorrectly simulated eye rotations by failing to incorporate dynamic perspective cues. More recently, heading selective neurons in the ventral intraparietal (VIP) area were reported to show rotation-tolerant heading tuning for properly simulated rotations (Sunkara et al., 2015). However, it remains unclear whether these visual compensation mechanisms benefit from rich depth structure in the scene. Our first main goal was to evaluate this question by recording neural activity in macaque area MSTd, which has been implicated in representing heading based on optic flow and vestibular signals (Tanaka et al., 1989; Duffy and Wurtz, 1995; Britten and van Wezel, 1998; Angelaki et al., 2011). To assess the role of depth structure, we simulated translation toward a 2D frontoparallel wall of random dots that contained dynamic perspective cues or translation through a 3D cloud of dots that contained motion parallax and disparity cues, in addition to dynamic perspective cues (Fig. 1*B*). To our knowledge, only one previous study (Yang and Gu, 2017) has systematically compared the rotation tolerance of heading tuning for 3D and 2D visual environments, using real pursuit eye movements. While that study did not find a clear effect in MSTd, the authors noted that 3D cues may have a greater effect when rotation is visually simulated. Thus, we examined the effect of depth cues for both real and simulated eye rotations.

During natural locomotion, translational self-motion is also accompanied by vestibular stimulation. It is well established that vestibular signals contribute to the precision of heading discrimination (Fetsch et al., 2009; Butler et al., 2010) and help to dissociate self-motion and object motion in both perception (Fajen and Matthis, 2013; Dokka et al., 2015a, 2019) and neural responses (Kim et al., 2016; Sasaki et al., 2017, 2019, 2020). Thus, we reasoned that vestibular translation signals might also contribute to rotation-tolerant heading tuning, which has not been addressed previously. Thus, the second major goal of this study was to test whether the heading tuning of MSTd neurons shows increased rotation tolerance when vestibular translation signals are added to optic flow.

## Materials and Methods

### Subjects, surgery, and apparatus

Data were collected from two adult male rhesus monkeys (*Macaca mulatta*) with average weights of 10.4 and 14.5 kg over the period of study. The monkeys were chronically implanted with a circular molded, lightweight plastic ring for head restraint, a recording grid, and a scleral coil for monitoring movements of the right eye. After recovering from surgery, the monkeys were trained using standard operant conditioning to fixate and pursue a visual target for liquid reward while head restrained in a primate chair. All surgical materials and methods were approved by the IACUC and were in accordance with National Institutes of Health guidelines.

The primate chair was fastened inside of a field coil frame (CNC Engineering) that was mounted on top of a six-degree-of-freedom motion platform (MOOG 6DOF2000E; Moog). A flat projection screen faced the monkey, and the sides and top of the field coil frame were covered with a black matte enclosure that restricted the animal’s view to the display screen. A stereoscopic projector (Christie Digital Mirage S + 3K) was used to rear-project images onto the 60x60cm display screen located ∼34.0 cm in front of the monkey, thus subtending almost 90° × 90° of visual angle. An OpenGL accelerator board (nVidia Quadro FX 4800) was used to generate visual stimuli at 1280 × 1024-pixel resolution, 32-bit color depth, and a refresh rate of 60 Hz. Behavioral control and data acquisition were controlled by custom scripts written for the TEMPO Experiment Control System (Reflective Computing).

### Stimulus, task, and cell selection

#### Stimulus

The visual stimulus was presented for 1500 ms during each trial and consisted of a random dot pattern that simulated various combinations of translation within the horizontal plane and eye rotation about the yaw axis (Fig. 1*A*,*C*). Translation along a straight path in one of eight evenly spaced directions (0° rightward, 45°, 90° forward, 135°, 180°, 225°, 270°, 315°) followed a trapezoidal velocity profile with a constant 23.1 cm/s velocity over the middle 750 ms and a total displacement of 26 cm (Fig. 1*D*, left). For conditions involving pursuit eye movements (real rotation), eye rotation was either leftward or rightward starting from a target location along the horizontal meridian that was ±8.5° from center, respectively, at the beginning of the trial. For simulated eye rotations, the fixation target remained centered on the display while the rotational component of optic flow simulated pursuit eye movements to the right or left. Rotation velocity also followed a trapezoidal profile, with sustained speeds of 15.1°/s for real rotation and 22.8°/s for simulated rotation during the middle 750 ms (Fig. 1*D*, right). Translational and rotational velocity profiles accelerated and decelerated during the first and last quarter of the trial, respectively. Because of a programming error that was discovered after experiments were completed, the rotation velocities for real and simulated eye rotations were not the same; thus, we refrain from making any direct comparisons between real and simulated eye rotation conditions. However, this issue did not affect real or visually simulated translations. All comparisons reported here are unaffected by this mismatch between real and simulated rotation velocities.

Optic flow stimuli were generated using a 3D rendering engine (OpenGL) to simulate combinations of observer translation and eye rotation. Rendering of optic flow was achieved by placing an OpenGL “camera” at the location of each eye and moving the cameras through the 3D-simulated environment along the same trajectory as the monkey’s eyes.

The visual stimulus was either a 3D cloud of dots or a 2D frontoparallel plane of dots (Fig. 1*B*). Each dot was a randomly oriented, 2D equilateral triangle with a base of 0.15 cm. In the 3D cloud stimulus, the random-dot pattern was 150 cm wide, 150 cm tall, 120 cm deep and had a density of 0.003 dots/cm^3^. To ensure that the depth range of the volume of dots visible to the monkey was constant during the 26-cm translation, near and far clipping planes were implemented such that dots were visible in the range from 10 to 80 cm from the observer. The 3D cloud was rendered as a red-green anaglyph that the monkey viewed stereoscopically through red-green filters (Kodak Wratten2, #29 and #61). At a viewing distance of ∼34 cm, binocular disparities ranged from −15° to +3.8° across the two animals (with slight variation because of different interocular distances). The 2D frontoparallel plane stimulus (150 × 150 cm) was rendered with a density of 0.5 dots/cm^2^ and zero binocular disparity, roughly matching the parameters used by Sunkara et al. (2015).

To increase the useful range of motion of the platform, the starting point of each translation was shifted in the direction opposite to the upcoming movement by half of the motion amplitude (failure to incorporate this offset properly led to the mismatch in rotation velocity between real and simulated rotation conditions). During forward translation, for example, the 26-cm displacement started 13 cm behind the center point of the motion platform’s range and ended 13 cm in front. For the 2D plane stimulus, this resulted in the simulated distance of the 2D wall from the observer changing from 47.0 cm at the beginning to 21.0 cm at the end of a trial. All other experimental parameters were the same between the 3D and 2D visual conditions.

Vestibular cues to translation were created by moving the motion platform with the same direction and velocity profile as the simulated translation conditions described above. Note that the platform, head fixed monkey, eye coil frame, projector, and display screen all moved together, such that the screen boundaries remained fixed relative to the head and body. Care was taken to ensure synchrony between visual and vestibular motion.

#### Task

The position of one eye was monitored online using an implanted scleral search coil. Liquid reward was given on trials in which the monkey’s gaze remained within a pre-determined electronic window. Trials were immediately aborted if the eye position fell outside of the window. Rotational optic flow was generated on the retina either by simulating eye rotation during central fixation (simulated rotation) or by requiring active pursuit of a moving fixation point (real rotation). During real rotation, the monkey was required to pursue a target that moved leftward or rightward on the screen, and needed to maintain eye position within an electronic window that was 4° × 4° during acceleration and deceleration of the pursuit target and 2° × 2° during the middle 750 ms of constant velocity target motion (Fig. 1*D*, right). The pursuit target, projected onto the display with zero binocular disparity, moved across the simulated translational flow field at a fixed viewing distance. Thus, in the real rotation condition, the rotational component of optic flow is produced by the eye’s rotation relative to the world. For the simulated rotation condition, the monkey fixated centrally within a window that shrunk from 4° × 4° to 2° × 2° during the middle 750 ms, while rotational components of optic flow were visually simulated by rotating the OpenGL cameras.

The optic flow stimulus was windowed with a software rendering aperture that moved together with the pursuit target. This ensured that the area of the visual field being stimulated during real pursuit trials remained constant over time. This method eliminated potential confounds that could be associated with the boundaries of the stimulus moving relative to the receptive field.

#### Cell selection

We included in this study any MSTd neuron that exhibited a well-isolated action potential (sorted online using a dual voltage-time window discriminator) and that met two additional criteria based on preliminary tests. First, a patch of drifting dots was presented for which the size, position, and velocity could be manually manipulated to map the receptive field and response properties of the neuron. Neural responses were required to be temporally modulated by a flickering patch of moving dots centered on the receptive field. Second, we ran a heading-tuning protocol that translated the monkey in the same eight heading directions within the horizontal plane as described above, while the monkey maintained central fixation. Three translation-only conditions (vestibular, visual, and combined) were used to determine the heading tuning of the neuron, with the visual and combined conditions involving simulated motion through a 3D cloud of dots. Neurons that showed significant tuning to heading in at least one of the translation-only conditions were included in our sample (ANOVA, *p* < 0.05).

### Experimental protocols

Two experimental protocols were used to manipulate different sets of variables. The depth variation protocol varied visual depth cues within the virtual environment while the vestibular variation protocol varied the presence or absence of vestibular cues to translation. Otherwise, the two protocols were the same in other respects.

#### Depth variation protocol

For the depth variation protocol, the virtual environment was randomly varied between the 3D cloud and the 2D frontoparallel plane (Fig. 1*B*). Translational self-motion was visually simulated in one of eight headings (Fig. 1*C*) and was combined with real or simulated eye rotation in both leftward and rightward directions. Thus, there were 64 distinct stimulus conditions that involved translation and rotation: [two rotation types: real/simulated] × [two directions of rotation] × [eight directions of translation] × [two virtual environments: 3D/2D]. In addition, to measure neural responses to pure translation based on visual and vestibular cues, we also interleaved translation-only control conditions. For each of the eight headings, responses to pure translation were measured by translating the motion platform while the visual display was blank except for a fixation target (vestibular translation), by translating the motion platform with a congruent visual stimulus (combined translation), or by simulating translation on a stationary platform (visual translation). The latter two conditions involving optic flow were presented twice, once with a 3D cloud and once with a 2D wall for the virtual environment. Thus, there were 40 translation-only control conditions: [eight headings] × [five translation conditions]. Self-motion in these control conditions had a trapezoidal velocity profile identical to that described above (Fig. 1*D*, left).

To measure neural responses to pure rotation, we also interleaved rotation-only control conditions including leftward and rightward real rotation with a blank background (we refer to this as “dark rotation” although the environment was not completely dark because of background illumination of the projector), and both real and simulated rotation with 3D cloud and 2D wall backgrounds. Thus, there were 10 rotation-only control conditions: [two rotation types (real, simulated)] × [two rotation directions (left, right)] × [two environments (3D, 2D)] + [two rotation directions in darkness]. In total, the depth variation protocol included 114 randomly interleaved stimulus conditions (64 translation/rotation, 40 translation-only, 10 rotation-only) plus a fixation-only condition to measure spontaneous activity with a blank background.

#### Vestibular variation protocol

The presence or absence of vestibular heading signals was manipulated to measure the contribution of vestibular signals to rotation compensation in MSTd. In the vestibular variation protocol, translational self-motion was either visually simulated by optic flow (visual only) or presented as a congruent combination of optic flow and real translation of the motion platform (combined), and these two translation types were combined with either real or simulated eye rotation. This protocol only used the 2D wall virtual environment. Thus, there were again 64 translation/rotation conditions in this protocol: [two rotation types] × [two directions of rotation] × [two translation types] × [eight directions of translation]. The same translation-only and rotation-only conditions as described above for the depth variation protocol were also included in this protocol, but without the control conditions that used the 3D cloud environment. Thus, there were 24 translation-only conditions and six rotation-only conditions, making a total of 94 randomly interleaved stimulus conditions plus a fixation-only null condition.

#### Protocol selection

For each of the above protocols, stimulus conditions were randomly interleaved and each condition was repeated three to seven times, with most recordings having five repetitions. Both protocols were designed to be run independently, and on many occasions, we were able to run both protocols on the same cell because of stable isolation. Once a cell was isolated, if it had significant vestibular tuning, the vestibular variation protocol took precedence. Otherwise the first protocol to be run was chosen pseudo-randomly. The vestibular variation protocol was run first in 48% of sessions that involved both protocols. Whenever possible, the second protocol was also run. Because of having some duplicate conditions between the two protocols, if there was doubt that the monkey would continue to work for the entire second protocol, an abbreviated version of the second protocol was used that eliminated some or all of the duplicate conditions. For example, both protocols included translation-only and rotation-only control conditions in the 2D environment which accounted for 24 and 6 conditions, respectively. Both protocols also contained the combined translation (simulated) and rotation (real and simulated) conditions in the 2D environment but in most cases, these conditions were retained when running both protocols. In total, 20% of cells were run only on the full-depth variation protocol, 21% of cells were run only on the full vestibular variation protocol, and the remaining 59% of cells were run on both protocols, the second of which may or may not have included duplicate conditions. In all cases for which both protocols were run on the same cell, the data from the two protocols were merged offline as long as there were at least three complete repetitions for each protocol. This resulted in some conditions having a different number of completed repetitions than others in cases where a condition was present in both protocols or when the number of repetitions within each protocol differed.

### Electrophysiological recordings

Extracellular single unit activity was recorded from one hemisphere of each monkey (left hemisphere of monkey A, right hemisphere of monkey C) using tungsten microelectrodes with a typical impedance in the range of 1–3 MΩ (FHC Inc.). At the start of each session, a sterile microelectrode was loaded into a custom made transdural guide tube and was advanced into the brain using a hydraulic micromanipulator (Narishige). The voltage signal was amplified and filtered (1–6 kHz, BAK Electronics). Single unit spikes were detected using a window discriminator (BAK Electronics) and recorded at 1-ms resolution. Eye position signals were sampled at 1 kHz, downsampled and smoothed to an effective resolution of 200 Hz using a boxcar average, and stored to disk by TEMPO software (Reflective Computing). The raw voltage signal from the electrode was also digitized and recorded to disk at 25 kHz (Power 1401 data acquisition system, Cambridge Electronics Design).

Area MSTd was located using a combination of magnetic resonance imaging, stereotaxic coordinates, white and gray matter transitions, and physiological response properties. In some penetrations, anatomic localization of MSTd was confirmed by advancing electrodes past MSTd, through the quiet area of the superior temporal sulcus, and into the retinotopically organized area MT. The size and eccentricity of the MT receptive fields encountered after passing through putative MSTd helped to confirm the placement of our electrodes within the dorsal subdivision of MST.

### Analysis

Analysis of spike data and statistical tests were performed using custom software written in MATLAB (MathWorks). Heading tuning curves for different combinations of translation and rotation were generated using the average firing rate of each cell (spikes/s) during the middle 750 ms of each successfully completed trial. This analysis window captured the part of the trial in which rotational and translation velocities were constant and eye position was within the 2° × 2° window. The effect of eye rotation on neural responses was determined by quantifying the difference between translation-only tuning curves and tuning curves produced by combined translation and rotation.

#### Quantifying tuning curve transformations

**Figure 2.**
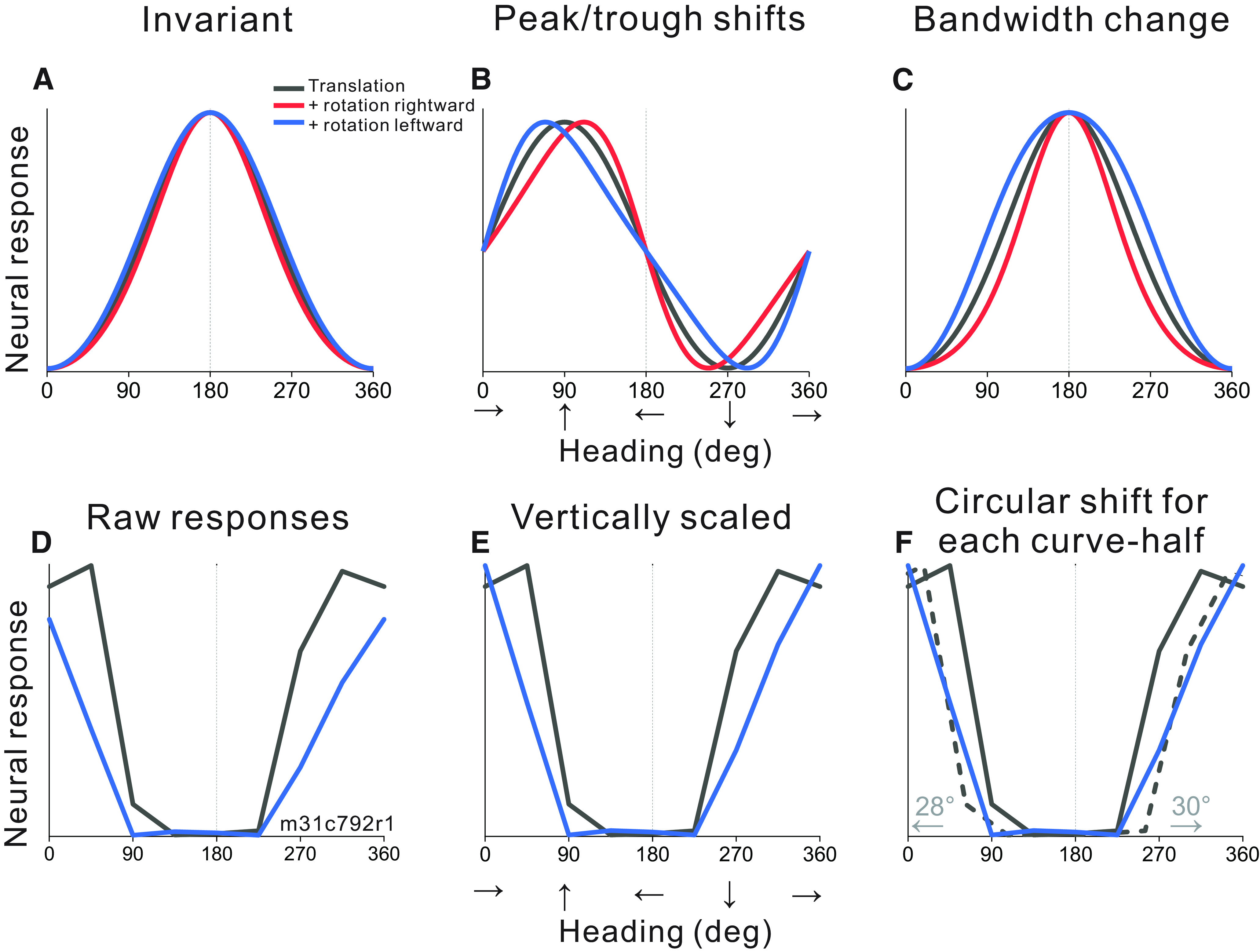
Quantifying the effect of eye rotation on heading tuning curves. ***A–C***, Schematic illustration of possible effects of eye rotation. Black curves represent responses to pure translation. Red and blue curves represent responses to combinations of translation and either rightward or leftward rotation, respectively. ***A***, Schematic illustration of complete compensation for eye rotations. ***B***, Schematic tuning of a cell with a forward heading preference (90°) that does not compensate for rotation, producing shifts of the peak and trough of the tuning curve in opposite directions. ***C***, Schematic tuning of a cell with a lateral heading preference (180°, leftward) that does not compensate for rotation resulting in changes in tuning bandwidth without a shift in the heading preference. ***D–F*,** Illustration of steps in the computation of partial shifts. ***D***, Tuning curves from a neuron responding to simulated translation and simulated rotation in the 2D environment. ***E***, Both tuning curves are linearly interpolated and the translation+rotation tuning curve (blue) is vertically scaled and shifted to match the range of responses in the pure translation curve (black). ***F***, Dashed lines indicate circularly shifted segments of the pure translation tuning curve that minimize the sum of squared error in each half of the translation+rotation tuning curve (0:180°, 180:360°). Partial shifts are indicated with arrows. Panels ***B***, ***C***, ***F*** show that the expected direction of the shift for each tuning curve half does not depend on heading preference.

A critical component of our analysis is the ability to distinguish between gain changes, bandwidth changes, and horizontal shifts of the tuning curve that are associated with the presence of visual or extraretinal eye rotation signals. This was possible because we sampled the full 360° range of headings. Previous studies in MSTd (Bradley et al., 1996; Page and Duffy, 1999; Shenoy et al., 1999, 2002; Maciokas and Britten, 2010) and VIP (Zhang et al., 2004; Kaminiarz et al., 2014) measured responses to a narrow range of headings, such that shifts in heading tuning were often indistinguishable from gain changes, bandwidth changes, or other changes to the shape of tuning. As a result, some previous studies suggested that eye rotations cause a global shift of heading tuning curves in the absence of pursuit compensation (Bradley et al., 1996; Page and Duffy, 1999; Shenoy et al., 1999, 2002; Bremmer et al., 2010; Kaminiarz et al., 2014). However, eye rotation can change the shape of heading tuning curves in ways that were not predicted by previous studies and can incorrectly appear as a shift within a narrow band of tuning (Fig. 2*A–C*; see also Sunkara et al., 2015).

To account for these more complex changes in heading tuning curves, we used a method that was developed to measure rotation compensation in a study of area VIP (Sunkara et al., 2015). Translation+rotation tuning curves were paired with translation-only tuning curves according to the translation type (visual or combined) and environment (3D or 2D). The first step in the analysis was to use the minimum and maximum responses from the translation-only tuning curve to vertically shift and scale the translation+rotation tuning curves to equate the range of responses between the curves (Fig. 2*D*,*E*). This corrected for any changes in gain that may result from eye rotation. Second, all tuning curves were linearly interpolated to 1° resolution and translation+rotation tuning curves were split into forward (0:180°) and backward (180:360°) ranges of headings, referred to as curve-halves. The interpolated translation-only curve was then circularly shifted in 1° increments to find the minimum sum-squared-error between the translation-only tuning curve and each translation+rotation curve-half (Fig. 2*F*); this defined the partial shift for each curve-half. Because some of our tuning curves were bimodal, we employed an additional step in this shift analysis. If the translation-only curve was categorized as bimodal (see Materials and Methods) and the partial shift was >90°, we searched for a local minimum closer to 0° or 360° in the sum squared error curve produced by the 360° circular shift. Finally, the sign of each partial shift value was adjusted so that positive values indicated shifts in the expected direction for cells that do not compensate for rotation. This analysis resulted in four partial shift values per neuron per condition: one for each half of the translation+rotation tuning curve for both right and left rotation conditions.

The individual partial shift values were accepted if they fulfilled three criteria. First, each non-interpolated translation+rotation curve-half and its non-interpolated translation-only tuning curve was required to have significant tuning (ANOVA, *p* < 0.05). Second, the bootstrap-derived confidence interval for the partial shift value was required to be no larger than 45°. This requirement eliminated unreliable shift values caused by poorly tuned curve-halves that passed ANOVA. Third, to eliminate partial shifts from tuning curve halves that had weak responses on one half of the curve, we only accepted partial shifts from curve halves with an average response amplitude at least one-half as large as that of the stronger curve-half. Amplitudes of the two curve halves were measured as the mean responses to forward headings (45°, 90°, and 135°) and backward headings (225°, 270°, and 315°).

In total, 34% of the partial shift values were eliminated. Accepted partial shift values were then averaged within each neuron and condition to quantify the ability of a single neuron to compensate for eye rotation within the condition. Rotation tolerance is therefore a result of rotation compensation which is measured by this shift metric. Across all conditions and neurons, 29.0% of the mean shifts were based on all four partial shifts, 10.1% were based on three partial shifts, 41.7% were based on two partial shifts, 8.5% were based on one partial shift, and 10.6% were eliminated because none of the partial shifts met all criteria. Extensive visual inspection of data was performed to verify that this set of criteria generally accepted reliable partial shift values; note, however, that no data were selected or excluded by visual inspection once the criteria were set and applied uniformly to all neurons. These criteria differ somewhat from the criteria employed in a study by Sunkara et al. (2015), which was necessary because more MSTd neurons had bimodal tuning curves or curves with weak responses to backward headings.

#### Expected shifts in the absence of compensation

The magnitude of translational flow vectors decreases with distance from the observer whereas the magnitude of rotational flow vectors is the same across all distances (Longuet-Higgins and Prazdny, 1980). Eye rotation therefore causes a larger shift of the FOE/FOC at greater distances where the rotational flow vectors have a greater effect on the global pattern of optic flow. This also means that the magnitude of shift during motion relative to a 2D frontoparallel wall will continually change over time while other parameters remain constant. For a forward translation and real eye rotation, the FOE shifts from 44° to 12° during the middle 750-ms analysis window. For simulated eye rotation and forward translation, the FOE comes into view at 960 ms from stimulus onset with a shift of 38° and decreases to 22° at the end of the analysis window. FOC shifts have the same magnitudes in the reverse order during backward translation. We averaged the succession of these values to approximate expected shifts of 26° and 28° for real and simulated eye rotation with the 2D stimuli, under the assumption that MSTd responses are driven solely by the resultant optic flow and do not compensate for rotation. Unlike the frontoparallel wall, the 3D cloud stimulus will have different shifts of the FOE/FOC for each depth plane at each moment in time, and the shifts increase in eccentricity with depth. The closest visible plane of the 3D cloud produced a shift of 7° with real rotation and became undefined ∼49% into the depth of the cloud. The computed shift at the closest plane on the 3D cloud during simulated rotation was 10° and became undefined 29% into the depth of the cloud. The shift at the closest plane can be considered a minimum estimate of expected shift for 3D stimuli under our null hypothesis. While these calculations provide some idea of how much tuning curves might shift in the absence of compensation, all of our main comparisons of interest are independent of the specifics of these calculations.

#### Detecting bimodal tuning curves

A subset of neurons in our population had bimodal heading tuning curves, as found previously in MSTd (Fetsch et al., 2007; Sato et al., 2013; Yang and Gu, 2017; Page and Duffy, 2018). Multiple peaks pose a challenge for our circular shift analysis (described above), since it is possible to reach minimum squared error by aligning to a peak that is up to 180° from the actual shift. To identify neurons with bimodal heading tuning, translation-only tuning curves were fit with unimodal and bimodal versions of a wrapped Gaussian function (Eqs. 1, 2) that were parameterized as follows:

(1) $$y_{uni}=a\;*\;e^{-2*\frac{1-\cos\left(\theta -\theta_{0}\right)}{\sigma_{uni}^{2}}}\;+\;R_{0}$$

(2) $$y_{bi}=a\;*\left(e^{-2*\frac{1-\cos\left(\theta -\theta_{0}\right)}{\sigma_{uni}^{2}}}\;+\;g\;*\;e^{-2*\frac{1-\cos\left((\theta -\theta_{0}\right)-\Delta )}{\sigma_{bi}^{2}}}\right)\;+\;R_{0},$$

where $\theta_{0}$ is the location of the primary/only peak, σ is the tuning width of each peak, *a* is the amplitude of the primary/only peak, *g* is the amplitude of the secondary peak relative to the primary peak, $R_{0}$ is baseline response, and *Δ* is the distance between the two peaks of the bimodal curve. The second exponential term in Equation 2 can produce a second peak out of phase with the first peak if parameter *g* is sufficiently large. Parameter bounds are summarized in Table 1.

**Table 1:** Parameters for tuning curve fits

Parameter	Lower bound	Upper bound
$\theta_{0}$ (°)	−360°	360°
σ_uni,_ σ_bi_ (°)	0.5°	10°
*A* (spk/s)	0	1.5 * response range
*g*	0	1
$R_{0}$ (spk/s)	0	Maximum response
*Δ* (°)	130°	230°

The log likelihood over the constrained parameter space was maximized for each tuning function to estimate each parameter (four parameters for the unimodal function, 7 for the bimodal function) using the *fmincon* function in MATLAB (MathWorks). Each curve was fit 200 times with each model while varying starting parameters and the best fit was chosen for each curve. The log likelihood ratio test was used to determine which of the two functions, unimodal or bimodal, was the better fit (χ^2^, *p* < 0.05). Bimodal classification also required that the amplitude of the secondary peak is at least 20% of the amplitude of the primary peak. Amplitudes were measured by subtracting the smallest response of the fitted bimodal curve from the response at the peaks.

#### Computing confidence intervals

A bootstrap analysis was used to calculate 95% confidence intervals on the tuning curve shift measurements. Bootstrapped tuning curves were generated by resampling single trial responses within each condition, with replacement (1000 iterations). The paired translation-only and translation+rotation tuning curves for each bootstrap iteration underwent the same shift analysis (described above) to measure the four partial shifts per condition. Each bootstrapped translation-only tuning curve was assigned the same modality classification (unimodal/bimodal) as the original curve. To measure the mean shift for each bootstrap iteration, partial shifts from curve halves that had significant tuning were averaged. This produced a distribution of 1000 mean shifts for each condition and for each neuron. The confidence interval was defined as the bounds of the middle 95% of the distribution (between the 2.5th and 97.5th percentiles).

#### Quantifying rotation selectivity

We analyzed data from the rotation-only control conditions to measure the selectivity of each neuron for pure rotation. The strength of selectivity for the direction of eye rotation (left vs right) was quantified by computing a direction discrimination index (DDI) from responses to rotation-only conditions (Prince et al., 2002; Uka and DeAngelis, 2003):

(3) $$DDI=\frac{\left|R_{r}-R_{l}\right|}{\left|R_{r}-R_{l}\right|\;+\;2\sqrt{\sigma_{r}^{2}+\sigma_{l}^{2}}},$$

where $R_{r}$ and $R_{l}$ are mean responses to rightward and leftward rotation, and $\sigma_{r}$ and $\sigma_{l}$ are the SDs of responses to rightward and leftward rotations, respectively. This produces DDI values between 0 (weak discrimination) and 1 (strong discrimination) for each rotation-only condition. DDI values were used to quantify the strength of the relationship between rotation tolerance and rotation selectivity in MSTd neurons.

#### Population decoding

We used an optimal linear estimator (OLE; Salinas and Abbott, 1994) to quantify the effects of depth cues and vestibular signals on heading estimates extracted from population activity in MSTd. Unlike the population vector algorithm (Georgopoulos et al., 1999), the OLE method is not affected by the nonuniform distribution of heading preferences known to exist in MSTd (Gu et al., 2010). It also does not strictly require cosine-like tuning curves, and precise decoding can be achieved with a smaller number of neurons than the population vector algorithm (Salinas and Abbott, 1994; Sanger, 1996; Georgopoulos et al., 1999; Schwartz et al., 2001). To comply with the requirements of linear decoding, all vectors in polar coordinates, specified by a heading direction and neural response, were converted to 2D Cartesian coordinates for the following computations.

Heading is optimally estimated by an OLE in a two-step process. The first step is to compute a set of weight vectors $\vec{D}$ that minimize the squared error between the estimated population vector and the true heading (the following methods are based on Salinas and Abbott, 1994). The weight vector ${\vec{D}}_{i}$ for neuron *i* is determined by

(4) $${\vec{D}}_{i}=\overset{n}{\underset{j=1}{\sum }}Q_{ij}^{-1}{\vec{L}}_{j},$$

where $Q_{ij}$ is the dot product of the tuning curves of neurons *i* and *j* unless *i* equals *j*, in which case the variance of neuron *i* is added to the dot product. ${\vec{L}}_{j}$ is the center of mass of the tuning curve of neuron *j* (for details, see Salinas and Abbott, 1994). The inputs used to produce our weight vectors $\vec{D}$ were (1) a list of firing rates averaged across repetitions for each neuron and for each heading condition during visually simulated translation-only trials; (2) a list of corresponding heading directions; and (3) a list of corresponding measures of neural response variance. Correlated noise is not considered in this analysis, as neurons were not recorded simultaneously.

In the second step, heading is decoded from population activity by calculating the population vector $\vec{V}$ for each condition *k*:

(5) $${\vec{V}}_{k}=\overset{N}{\underset{i=1}{\sum }}r_{ik}{\vec{D}}_{i},$$

where $r_{ik}$ is the firing rate of neuron *i* in heading condition *k*. The heading estimate ${\vec{V}}_{k}$, in 2D Cartesian coordinates, is transformed to polar coordinates where the heading angle is wrapped to the range [0°,360°].

To assess the uncertainty of the decoded estimates, we randomly resampled firing rates with replacement from within each simulated translation direction, resulting in 1000 bootstrapped repetitions per heading, per neuron. With eight headings (Fig. 1*C*), this results in 8000 bootstrapped trials per neuron. Bootstrapping was performed separately for each simulated rotation condition (left, right), and for the no-rotation condition which resampled trials used to train the OLE. Heading estimate ${\vec{V}}_{ki}$ was computed for each bootstrapped trial *i* using Equation 5, and the estimates from 1000 trials within each heading condition were averaged to produce one population vector estimate ${\vec{V}}_{k}$ per heading condition *k*. To measure the uncertainty of the population vector for each heading, we computed the 95% confidence intervals on the distribution of 1000 heading estimates using the percentile method. Unlike the other computations above, this was computed in polar coordinates since the heading estimates vary along the azimuthal plane rather than varying along the vertical and horizontal axes. Care was taken to ensure the angular conversion was wrapped to [0°,360°] bounds. If the distribution of heading estimates spanned the [0°,360°] bounds within a heading condition, all values were circularly shifted by 180° before computing the confidence interval range.

After establishing the weight vectors $\vec{D}$ from the visually simulated translation-only condition, decoding was performed separately for leftward, rightward, and no-rotation conditions using the same set of weight vectors $\vec{D}$. In other words, the weight vectors are computed to accurately estimate heading in the translation-only condition, and then the weights are applied to the translation+rotation conditions to predict biases in heading estimates caused by rotation. By comparing how biases in the estimates depend on depth cues and vestibular translation signals, we assess the effects of these cues on rotation compensation at the population level.

## Results

MSTd neurons were tested with two experimental manipulations: the depth structure of the visual environment was varied (depth variation protocol), or the sensory modality of the translational motion cues was varied (vestibular variation protocol). Either the depth variation protocol or the vestibular variation protocol (or both) were run on 101 isolated MSTd neurons from two monkeys (39 from the left hemisphere of monkey A and 62 from the right hemisphere of monkey C). Data from 19 neurons were eliminated from analysis because of not having at least three complete repetitions. We also required significant heading tuning (ANOVA, *p* < 0.05) for at least one of the translation-only tuning curves from either of the protocols, which eliminated data from another seven neurons. The analysis was therefore based on 75 neurons (28 from monkey A, 47 from monkey C).

**Figure 3.**
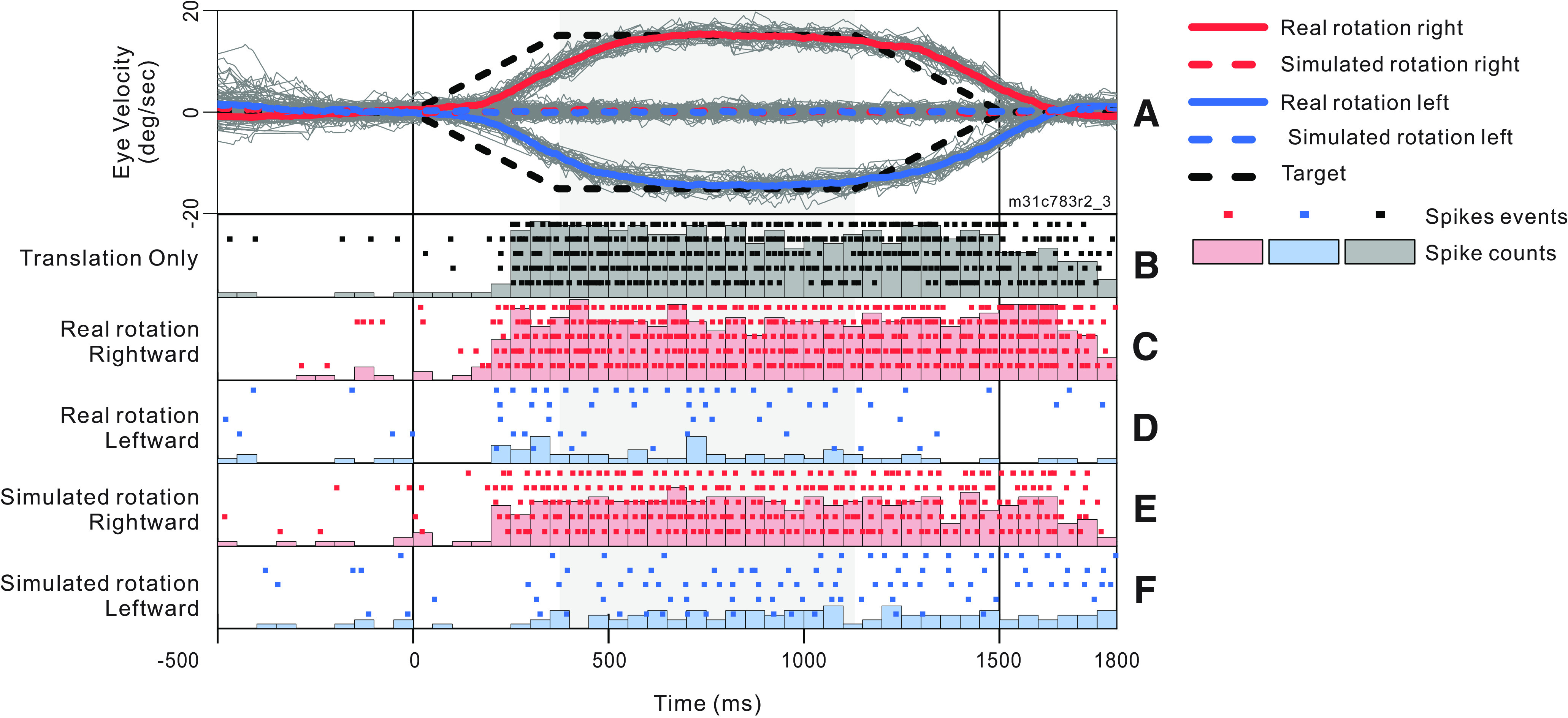
Example eye velocity traces and neural response histograms. Data were obtained during a recording from a single MSTd neuron (same cell as in Fig. 4*C*) in response to simulated translation in the 3D environment, combined with either real or simulated rotation. Vertical reference lines mark the start and end of the translation and rotation stimuli, while the shaded region indicates the analysis window. The animal maintained fixation of a target against a dark background for 500 ms preceding and 300 ms following the stimulus presentation. ***A***, Horizontal eye velocity traces from 160 individual trials (gray curves) are plotted along with average velocity traces for real and simulated rotations (solid and dashed thick curves, respectively) in left and right directions (blue and red, respectively). Eye position data were smoothed with a five-point moving average then differentiated. The resulting eye velocity signal was then smoothed with a five-point moving average. Saccades were identified by thresholding the acceleration signal; identified saccades were then removed and filled in by linear interpolation. The black, dashed line indicates target velocity. ***B–F***, Peristimulus time histograms and spike rasters showing neural responses during five repetitions of the preferred heading (90°) for the translation-only condition and the four translation+rotation conditions. PSTH heights range from 0 to 18 spikes per bin.

In each session, we recorded the spike trains of an MSTd neuron, along with eye movements (for details, see Materials and Methods). Figure 3*A* shows eye velocity traces for an example recording session. These eye traces were very typical, and demonstrate that the animal pursued the target quite accurately and reliably. The effect of catch-up saccades on neural responses was not analyzed systematically; however, effects of catch-up saccades were likely small given that the smooth eye velocity traces matched target velocity rather closely (Fig. 3*A*). Figure 3*B–F* shows responses from an exemplar neuron to stimuli presented at its preferred heading (90°) for the translation-only condition, as well as the four translation+rotation conditions. Strong response modulations related to the direction of real and simulated eye rotations are apparent. In subsequent figures, tuning curves were constructed from firing rates computed during the constant-velocity period (Fig. 3, gray shading).

### Effects of eye rotation on optic flow and expected effects on heading tuning

Eye rotation alters the retinal velocity pattern created by translational self-motion and offsets the FOE/FOC on the retina such that it no longer corresponds to the true heading (Fig. 1*A*). If the response of MSTd neurons is determined solely by translational velocity (heading), tuning curves obtained during real or simulated eye rotation should not differ appreciably from translation-only tuning (Fig. 2*A*). However, if the response is determined solely by the resultant optic flow on the retina, which reflects both translation and rotation, a distortion of the heading tuning curve is expected (Fig. 2*B*,*C*). Because rotation shifts the FOE and FOC in opposite directions (Fig. 1*A*, right), the heading tuning curve of a neuron that prefers forward translation would have a peak that shifts to the right (toward leftward headings) and a trough that shifts to the left (toward rightward headings) during rightward rotation (Fig. 2*B*, red curve). For the same neuron, leftward eye rotation would cause the peak to shift to the left (toward rightward headings) and the trough to shift to the right (toward leftward headings; Fig. 2*B*, blue curve). Neurons that prefer lateral headings, which are common in MSTd (Gu et al., 2010), are expected to primarily show changes in tuning bandwidth because of rightward and leftward rotations (Fig. 2*C*,*F*). Independent of preferred heading, heading representations are expected to shift inward toward 180° during rightward rotation and outward toward 0/360° during leftward rotation for our plotting scheme (Fig. 2*B*,*C*,*F*). Our null hypothesis is that neural responses are determined solely by the resultant optic flow on the retina and will produce translation+rotation tuning curves that deform as illustrated in Figure 2*B,C*. It is important to emphasize that the expected effect of rotation on heading tuning is not simply a global shift of the pure-translation tuning curve, as was previously assumed in studies that examined tuning over a narrow range of forward headings (Bradley et al., 1996; Shenoy et al., 1999, 2002).

### Effect of depth cues on rotation compensation in single neurons

**Figure 4.**
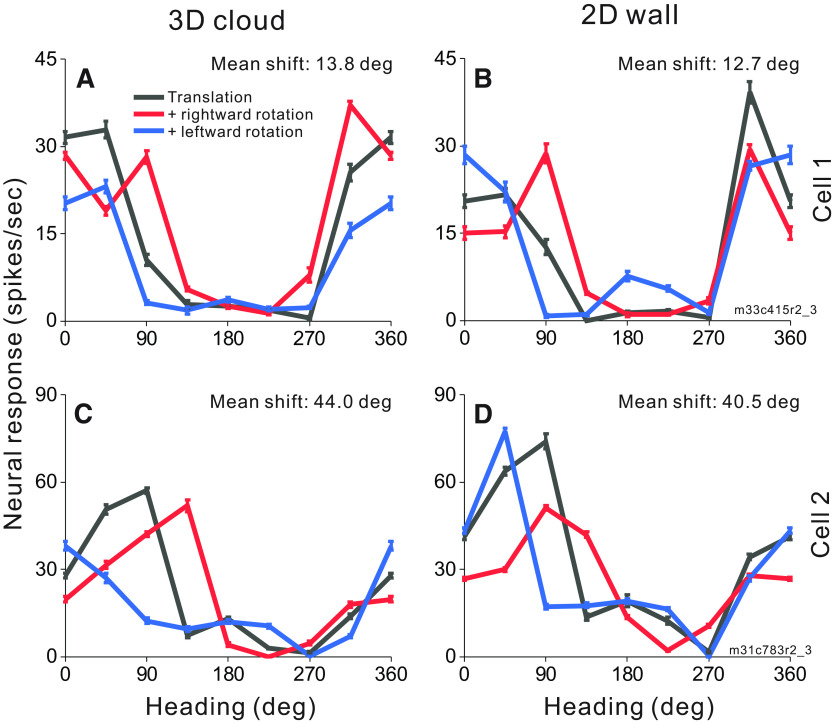
Heading tuning curves from two example MSTd neurons (rows) in the 3D and 2D environments (columns). ***A***, ***B***, Data from an MSTd neuron recorded during simulated translation and simulated eye rotation. Black curves show responses to pure translation during central fixation. Red and blue curves show responses to combinations of translation and rightward and leftward eye rotation, respectively. Error bars show standard errors of the mean. Mean shifts for the 3D cloud condition (***A***) and the 2D wall condition (***B***) are indicated above the respective tuning curves. ***C***, ***D***, Data from a second MSTd neuron, also during simulated translation and rotation.

To investigate whether MSTd neurons make use of motion parallax cues available in a 3D environment to compensate for rotation, we measured heading tuning during real or simulated eye rotation in two virtual environments: a 2D frontoparallel wall that affords dynamic perspective cues and a 3D cloud that affords both dynamic perspective and local motion parallax cues. Heading tuning curves measured during eye rotation are compared with translation-only tuning to determine whether a neuron’s response is driven primarily by translational velocity or reflects resultant optic flow. Figure 4 shows responses of two MSTd neurons to combinations of simulated translation and rotation for virtual environments corresponding to a 3D cloud (Fig. 4*A*,*C*) and a 2D wall (Fig. 4*B*,*D*). Cell 1 (Fig. 4*A*,*B*), which prefers nearly rightward heading in the translation-only condition (black), demonstrates changes in tuning bandwidth during simulated leftward (blue) and rightward (red) rotation with a weaker effect for backward headings in the 2D environment. This change of bandwidth is expected for cells that prefer lateral motion and do not fully compensate for eye rotation (Fig. 2*C*,*F*). The mean shifts for this cell are 13.8° and 12.7° for the 3D and 2D environments, respectively. Cell 2 (Fig. 4*C*,*D*), which prefers forward translation in the translation-only condition (black) shows clear shifts of the peak of the tuning curve for rightward and leftward rotations, in the directions expected for a neuron that does not compensate for rotation (Fig. 2*B*). The mean shifts are large for both the 3D (44.0°) and 2D (40.5°) environments. For both example neurons, tuning shifts are not smaller for the 3D environment than the 2D environment, suggesting that MSTd neurons may not benefit from the depth structure of the environment when responding to combinations of translation and rotation.

**Figure 5.**
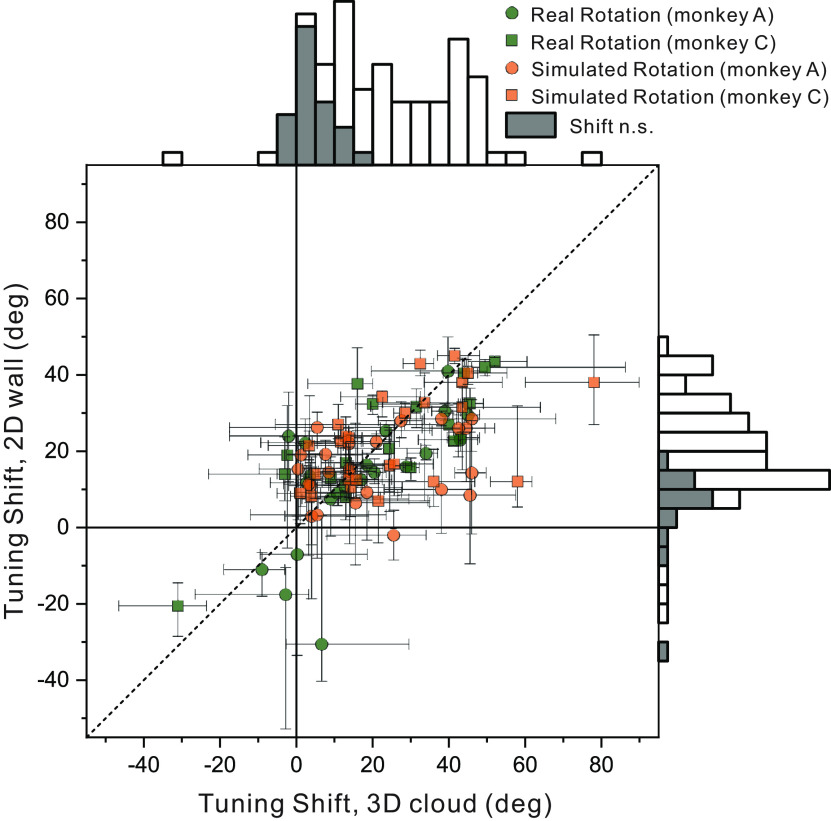
Summary of effects of depth structure on rotation compensation of MSTd neurons. The mean tuning shift for each neuron in the 3D (*x*-axis) and 2D (*y*-axis) environments is shown for conditions involving simulated translation combined with either real rotation (green) or simulated rotation (orange; 88 pairs of average tuning shifts from *N* = 47 neurons). Circles and squares denote data for monkeys A and C, respectively. Error bars depict bootstrapped 95% confidence intervals for each neuron/condition. Shaded bars in the marginal histograms represent neurons with shifts that are not significantly different from zero.

Across our population of 58 MSTd neurons tested in the depth variation protocol, we found a range of rotation compensation, including cells that show nearly complete compensation, cells that show little compensation for eye rotation, and a range of partial compensation (see Discussion). Figure 5 compares tuning shifts between the 3D cloud and 2D wall virtual environments. Because of our criteria for accepting reliable partial shifts (see Materials and Methods), Figure 5 contains data from 47 of the 58 neurons tested, resulting in 88 pairs of mean shift values (2D, 3D pairs) that met our selection criteria across the real and simulated rotation conditions. A shift of 0° indicates complete compensation for eye rotation, allowing the neuron to signal heading with invariance to rotational optic flow. Based on bootstrapped 95% confidence intervals, 16 cells have shifts that are not significantly different from zero for the 3D environment (seven cells for real rotation, one cell for simulated rotation, and eight cells for both rotation conditions), as well as 15 cells for the 2D environment (12 cells for real rotation, two cells for simulated rotation, and one cell for both conditions). Eight cells had shifts that were not significantly different from zero in both 3D and 2D environments (seven cells for real rotation, one cell for simulated rotation). Neurons that respond solely to the resultant optic flow on the retina are expected to shift by ∼26–28° for our 2D stimulus and a minimum of 7–10° for the 3D stimulus (see Materials and Methods). Shift values that fall along the unity-slope diagonal are affected by rotation equally for the 2D and 3D environments. The median shifts across the population (18.5° for 3D and 18.0° for 2D environments) do not differ significantly (Wilcoxon signed-rank test; *z* = 1.52, *p* = 0.128) between environments.

To test for an effect of depth structure (3D vs 2D) while controlling for differences across animals and rotation conditions, we performed a two-way repeated measures analysis of variance, with rotation type (real or simulated) and monkey identity (A or C) as cofactors. The main effect of depth structure again did not reach significance (*F*_(1,84)_ = 3.23, *p* = 0.076) and there were no significant interactions with monkey identity (*F*_(1,84)_ = 0.13, *p* = 0.724) or rotation type (*F*_(1,84)_ = 1.23, *p* = 0.271). Note also that the weak tendency was for tuning shifts to be greater in the 3D condition than the 2D condition (Fig. 5), which is opposite to the hypothesis that motion parallax cues would improve rotation compensation. Thus, we find no evidence, at the single-unit level in MSTd, that a richer depth structure containing local motion parallax leads to more stable heading tuning in the presence of eye rotations.

### Effect of vestibular translation signals on rotation compensation in single neurons

The instantaneous retinal flow field during self-motion reflects the combination of translational and rotational velocity of the eye in space. To help in isolating the translational component of self-motion, the brain might make use of translational vestibular signals that initially arise from the otolith afferents of the vestibular system (Angelaki and Cullen, 2008). To examine this idea, we tested 60 MSTd neurons in the vestibular variation protocol, which compared real and simulated translation. On real translation trials, a motion platform moved the animal along the same translational trajectories that were simulated by optic flow in the other conditions (Fig. 1*C*,*D*). If vestibular heading signals aid in the computation of rotation-invariant heading, we expect smaller shift values for the real translation condition relative to the simulated translation condition.

**Figure 6.**
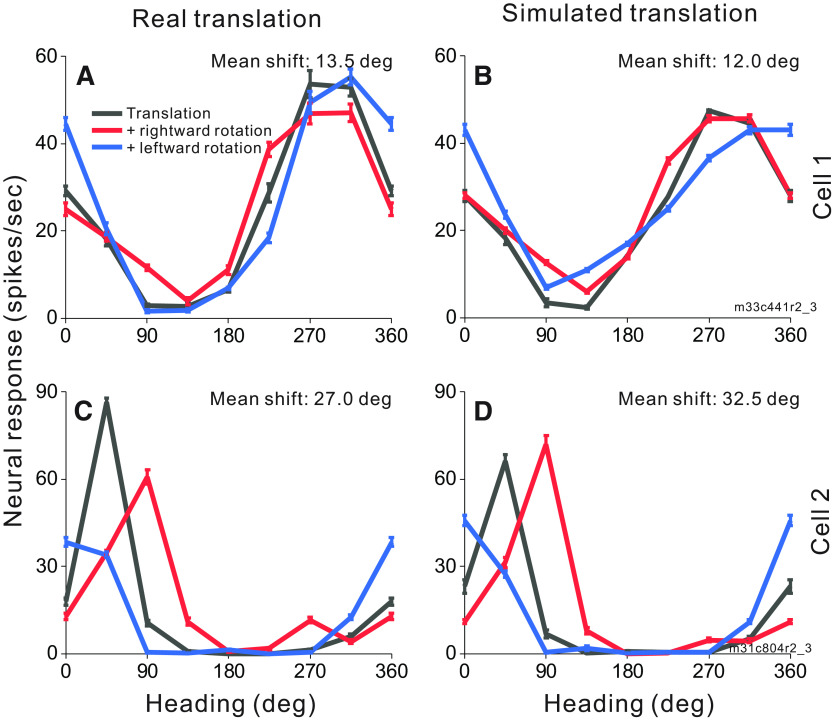
Heading tuning curves from two example MSTd neurons (rows) in the real and simulated translation conditions (columns). Figure conventions as in Figure 4. Both example cells were recorded during real translation (***A***, ***C***) and simulated translation (***B***, ***D***) combined with simulated eye rotation.

Figure 6 shows responses of two MSTd neurons to combinations of simulated rotation with real (Fig. 6*A*,*C*) and simulated (Fig. 6*B*,*D*) translation within the 2D frontoparallel wall environment. Cell 1 (Fig. 6*A*,*B*) prefers nearly backward headings in the translation-only condition (black) with small changes to the tuning curve during rightward (red) and leftward (blue) simulated rotation. The mean shifts for this cell are 13.5° and 12.0° in the expected direction for real and simulated translation, respectively. Cell 2 (Fig. 6*C*,*D*) prefers headings in the forward-rightward direction in the translation-only condition (black) but shows clear shifts of tuning curve peaks in simulated rotation conditions (red and blue) following the expectations for non-compensatory cells in Figure 2*B*. The mean shifts are large for both real translation (27.0°; Fig. 6*C*) and simulated translation (32.5°; Fig. 6*D*), indicating that the cell’s responses are mainly driven by resultant optic flow.

**Figure 7.**
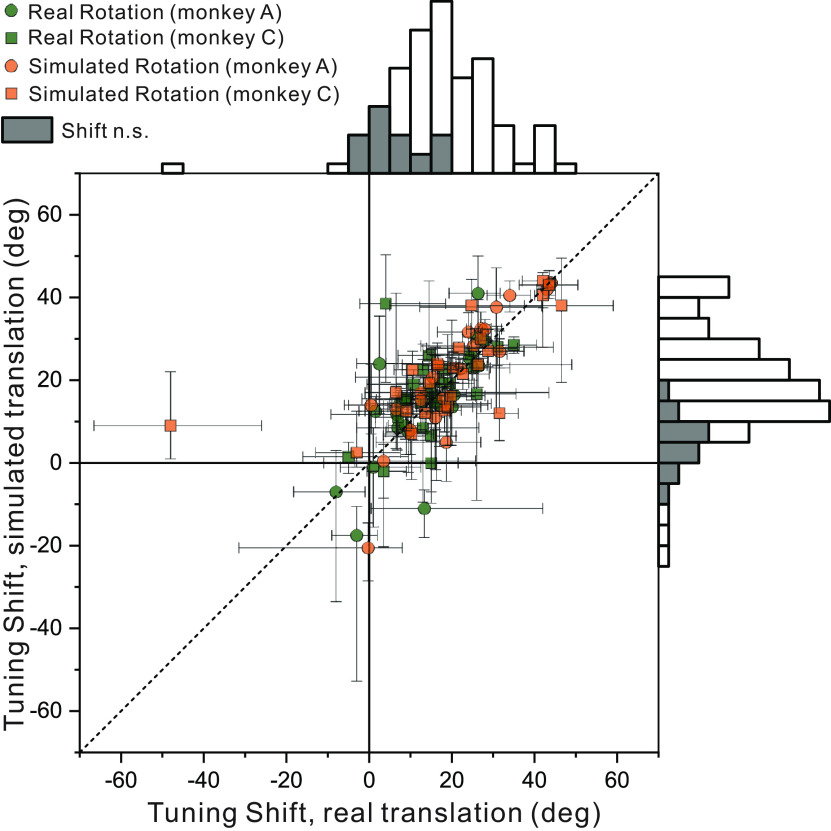
Summary of the effect of vestibular translation signals on rotation compensation for MSTd neurons. Tuning shifts are compared for real translation (*x*-axis) and simulated translation (*y*-axis) conditions. Data are shown separately for both real rotation (green) and simulated rotation (orange) conditions in the 2D wall environment (91 pairs of average shifts from *N* = 49 cells). Circles and squares denote data for monkeys A and C, respectively. Error bars depict bootstrapped 95% confidence intervals. Shaded bars in the marginal histograms represent cells with tuning shifts not significantly different from zero.

Figure 7 compares tuning shifts for each neuron between the real and simulated translation conditions. Because of elimination of unreliable partial shifts (see Materials and Methods), Figure 7 displays data from 49 of the 60 neurons tested, yielding 91 pairs of mean shift values that met our selection criteria. Unlike Figure 5, all data in Figure 7 come from the 2D wall environment. Based on bootstrapped 95% confidence intervals, 16 cells have shifts that are not significantly different from zero for real translation (eight cells for real rotation, three cells for simulated rotation, and five cells for both rotation conditions), and 13 cells have shifts not significantly different from zero for simulated translation (nine cells for real rotation, two cells for simulated rotation, and two cells for both rotation conditions). Six cells had shifts that were not significantly different from zero in both translation conditions (four cells for real rotation, 2 cells for both rotation conditions). Median shifts across the population for real and simulated translation were 16.3° and 17.0°, respectively, and did not differ significantly (Wilcoxon signed-rank test; *z* = 1.69, *p* = 0.090). To control for variations in rotation type (real or simulated) and monkey identity (A or C), we again performed a two-way repeated measures ANOVA. The main effect of translation type (real vs stimulated) was not significant (*F*_(1,87)_ = 2.15, *p* = 0.146) and there were no significant interactions with monkey identity (*F*_(1,87)_ = 0.696, *p* = 0.406) or rotation type (*F*_(1,87)_ = 0.123, *p* = 0.727). Thus, across the entire sample of MSTd neurons, we do not find that vestibular translation signals significantly enhance the rotation tolerance of heading tuning.

We further considered whether the effect of vestibular signals on rotation tolerance might depend on whether neurons show significant vestibular heading tuning in the absence of optic flow. Thirty-two of the 60 neurons in the vestibular variation protocol were significantly tuned for heading based solely on vestibular stimulation (ANOVA, *p* < 0.05). For this subset with significant vestibular tuning, median tuning curve shifts were 13.3° and 17.0° for real and simulated translation, respectively, and this difference was marginally significant (Wilcoxon rank-sum test; *z* = 2.02, *p* = 0.044). For the remaining 28 MSTd neurons without significant vestibular heading tuning, median tuning shifts were 19.3° and 16.6° for real and simulated translation, respectfully, and were not significantly different (*z* = 0.17, *p* = 0.867). Thus, for the subpopulation of MSTd neurons with significant vestibular tuning, we found modest evidence that vestibular translation signals may play a role in compensating heading tuning for eye rotation.

### Effect of rotation selectivity on rotation compensation

**Figure 8.**
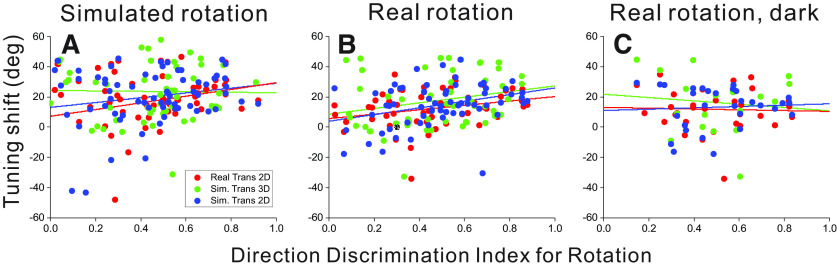
Summary of the relationship between rotation tolerance of heading tuning and selectivity for pure rotation. Rotation selectivity was quantified using a DDI (*x*-axis) and was compared with the mean shift (*y*-axis) for each cell. Data are shown separately for simulated rotation (***A***), real rotation across stationary background dots (***B***), and real rotation in darkness (***C***). Red, green, and blue points represent real translation in the 2D environment, simulated translation in the 3D environment, and simulated translation in the 2D environment, respectively. Trend lines show the least squares linear regression between DDI and mean shift for each condition (ANCOVA).

A broad range of rotation tolerance is evident across the population of MSTd neurons represented in Figures 5, 7. We investigated the possibility that a neuron’s tolerance to rotation is related to the neuron’s selectivity for pure rotation. DDI values (see Materials and Methods) were computed as a measure of neural selectivity for real or simulated rotation. Real eye rotations were either performed by pursuing a target across a blank background or pursuing a target across a visual background of stationary dots, the latter of which generated rotational optic flow on the retina. For each cell, DDI values were paired with mean shift values according to the type of rotation (real or simulated) and virtual environment (2D or 3D). DDI values from real eye rotation in darkness were paired with shift values from real rotation conditions in both 2D and 3D environments. The relationship between rotation tolerance and rotation selectivity was quantified for each pure-rotation type (real rotation across stationary dots, real rotation in darkness, and simulated rotation) using an analysis of covariance (ANCOVA) with DDI as a continuous variable and the translation/depth condition as a categorical factor with three levels (real translation in 2D, simulated translation in 2D and in 3D; Fig. 8). A positive slope between DDI and mean shift indicates that neurons with stronger rotation selectivity tend to have larger shifts and therefore less rotation tolerance.

Selectivity to pure visual rotation cues (simulated rotation) was compared with mean shifts from conditions that combined simulated visual rotation with translation, resulting in a weak main effect of rotation selectivity that approached significance (*F*_(1,167)_ = 3.44, *p* = 0.066; Fig. 8*A*). Neither the translation/depth factor nor the interaction between this factor and DDI were significant (*F*_(2,167)_ = 1.35, *p* = 0.26 and *F*_(2,167)_ = 0.91, *p* = 0.40, respectively). Rotation selectivity based on combined visual and extraretinal rotation cues (real rotation across stationary dots) was compared with mean shifts from conditions involving translation and real pursuit, resulting in a robust main effect of rotation selectivity (*F*_(1,166)_ = 13.43, *p* = 0.00033; Fig. 8*B*) and no significant main effect of translation/depth condition (*F*_(2,166)_ = 1.56, *p* = 0.21) or interaction (*F*_(2,166)_ = 0.19, *p* = 0.83). Finally, selectivity for real eye rotation in darkness was compared with mean shifts from conditions that combined translation and real eye rotation with optic flow in both 2D and 3D environments; this comparison did not result in any significant main effects or interaction in the ANCOVA model (*F*_(1,76)_ = 0.049, *p* = 0.83 for the main effect of DDI; Fig. 8*C*).

These results demonstrate that neurons with stronger rotation tolerance show weaker selectivity for pure rotation, at least for rotation based on optic flow (see Discussion).

### Effect of depth structure and vestibular translation signals on rotation compensation across the population

Thus far, we have examined effects of rotation on heading tuning at the level of single neurons. Since results across neurons are somewhat diverse and it is possible that rotation compensation could be achieved by selectively weighting the responses of subsets of neurons, we have also examined how rotation affects estimates of heading derived from population activity. All 75 neurons from the analyses described above were potentially included in the population decoding analysis but some neurons were not exposed to all experimental conditions. This resulted in populations of 58 neurons for the depth cue comparison and 60 neurons for the vestibular condition comparison.

#### Heading decoding for 3D and 2D environments

**Figure 9.**
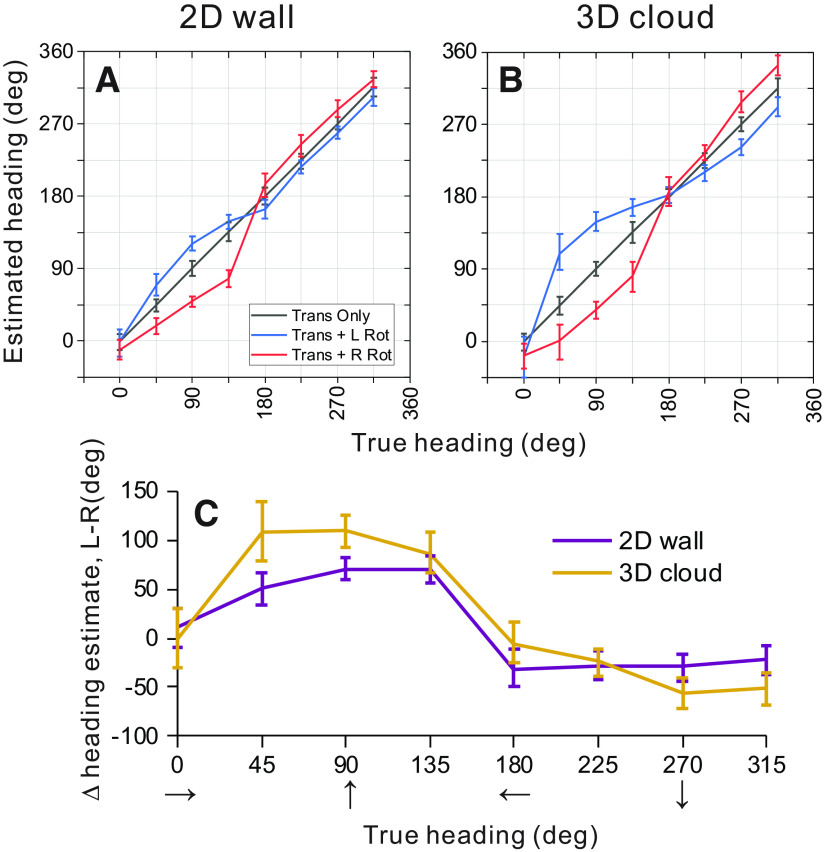
Summary of population decoding results for 2D and 3D environments. An OLE was used to decode heading from population responses to simulated translation and rotation conditions (see text for details). ***A***, ***B***, Weight vectors were computed separately for 2D (***A***) and 3D (***B***) environments from translation-only trials. Those weight vectors were then used to decode bootstrapped neural responses from translation-only (gray), translation plus rightward rotation (red), and translation plus leftward rotation (blue) conditions. Decoded heading estimates versus true headings are shown for the 2D (***A***) and 3D (***B***) environments. ***C***, Estimated headings for rightward rotation conditions were subtracted from estimated headings for leftward rotation conditions, and this difference is plotted as a function of true heading. Results are shown separately for the 2D (purple) and 3D (gold) depth environments. Error bars in all panels show 95% confidence intervals.

Heading was estimated from MSTd population activity using an OLE approach (Salinas and Abbott, 1994; for details, see Materials and Methods). For each depth cue condition, weight vectors $\vec{D}$ were computed from neural responses to the simulated translation-only condition (Eq. 4) and those weight vectors were then used to decode bootstrapped responses (Eq. 5) from the same translation-only condition (Fig. 9*A*,*B*, gray line), from the translation with simulated leftward rotation condition (Fig. 9*A*,*B*, blue line), and from the translation with simulated rightward rotation condition (Fig. 9*A*,*B*, red line).

In the absence of rotation, as expected, heading estimates produced by the OLE were very accurate for the 2D wall condition, with errors in mean heading estimates ranging from 0.17° to 0.70° (mean = 0.40°) and mean 95% confidence intervals of ±9.7° (Fig. 9*A*, gray line and error bars). Similarly, for the 3D cloud environment, errors in mean heading estimates for the translation-only condition ranged between 0.06° and 0.97° (mean = 0.33°) with mean confidence intervals of ±10.4° (Fig. 9*B*, gray line). The OLE algorithm is therefore capable of decoding heading quite accurately in the absence of rotation for both visual environments.

To make predictions of biases in heading estimates that are caused by rotational optic flow, the same weight vectors $\vec{D}$ (that were trained to decode translation-only conditions) were applied to responses from rotation-added conditions. The logic of this approach is as follows: we assume that decoding weights are optimized to estimate heading in the absence of rotation and that those same weights are applied when rotations are present. This approach resulted in patterns of substantial biases in the directions expected from incomplete rotation compensation (Fig. 9*A*,*B*). Heading errors are greatest around forward headings (45°, 90°, 135°) for both depth cue conditions, where the maximum heading errors were 30.7° and 63.7° for leftward rotation in the 2D and 3D environments, respectively (Fig. 9*A*,*B*, blue lines). For rightward rotation, the corresponding errors are 57.3° and 53.7° (Fig. 9*A*,*B*, red lines).

Heading estimates during rightward rotation were subtracted from heading estimates during leftward rotation to summarize the effect of eye rotation on the population response. Figure 9*C* shows that eye rotation generally had a slightly greater effect on population estimates of heading for the 3D cloud condition (gold) than for the 2D wall condition (purple); 95% confidence intervals on the heading errors show a significant difference between the depth cue conditions for headings of 90° (forward translation) and 45° (forward-right translation), whereas there is no significant difference between depth cue conditions for the remaining headings. These population results are consistent with the conclusions of our single-cell analysis (Fig. 5), in that the addition of 3D structure does not improve rotation tolerance, but actually makes it slightly worse. This is clearly inconsistent with the hypothesis that 3D cues (e.g., motion parallax) are important for creating tolerance to rotation, at least in MSTd.

#### Heading decoding for real versus simulated translation

**Figure 10.**
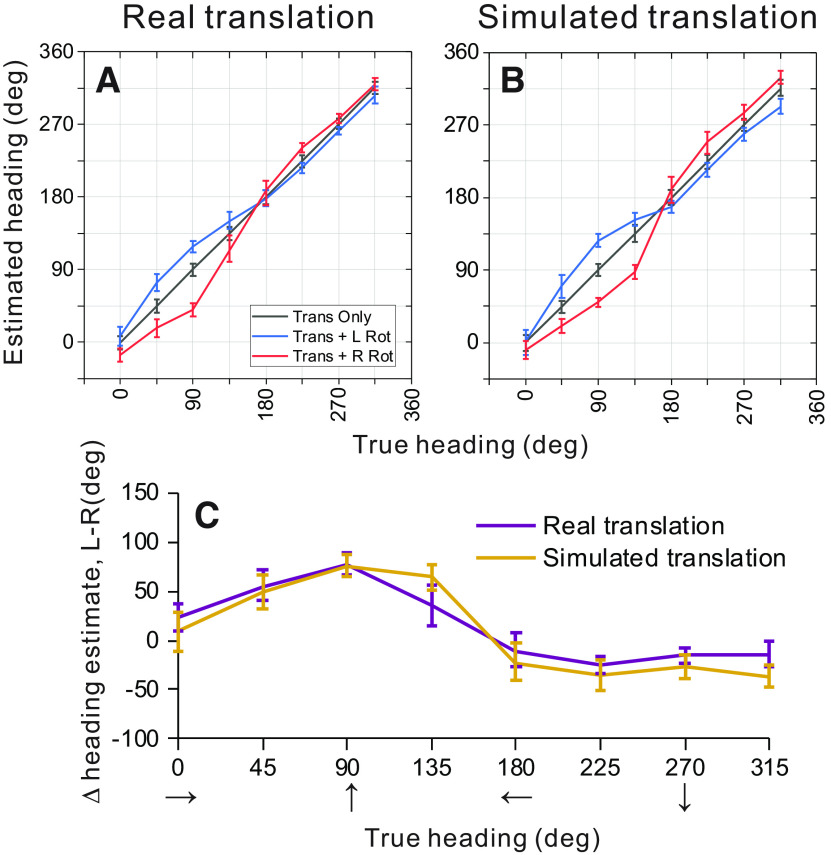
Summary of population decoding results for real versus simulated translation. ***A***, ***B***, Decoded heading estimates versus true headings are shown for the real and simulated translation conditions, respectively. ***C***, Differential heading biases between rightward and leftward rotations are plotted as a function of true heading. Figure conventions as in Figure 9.

Following the same procedure described above, weight vectors $\vec{D}$ were computed (Eq. 4) from neural responses to translation-only conditions in the 2D environment for each translation type (real and simulated). Since some recordings did not include all conditions, the population of neurons used for this analysis differs slightly from that used in the previous section. The weight vectors from each translation type were used to decode bootstrapped responses from within the same translation-only condition (gray), as well as from translation with simulated leftward rotation (blue) and translation with rightward rotation (red) conditions (Fig. 10*A*,*B*). The mean error of heading estimates produced by the OLE for real translation-only stimuli ranged from 0.004° to 0.51° (mean = 0.14°), with a mean 95% confidence interval of ±7.7° (Fig. 10*A*, gray). Similarly, for simulated translation, mean heading errors for the translation-only condition ranged from 0.001° to 0.55° (mean = 0.18°) with a mean confidence interval of ±8.8° (Fig. 10*B*, gray line). Again, OLE estimates heading for the translation-only conditions in a largely unbiased fashion.

The same weight vectors $\vec{D}$ were then used to decode responses from rotation-added conditions, which resulted in a similar pattern of heading errors as discussed in the previous section. For the real and simulated translation conditions, respectively, maximum deviations from true headings were 28.5° and 36.3° for leftward rotation (blue), and 49.9° and 47.7° for rightward rotation (Fig. 10*A*,*B*, red). Figure 10*C* summarizes the effect of rotation on population estimates for the real and simulated translation conditions. There were no headings for which 95% confidence intervals indicated a significant difference between the two translation conditions. This finding is consistent with the result of the single cell analyses in Figure 7, demonstrating that vestibular translation signals do not enhance rotation tolerance of heading tuning in area MSTd.

## Discussion

We investigated how heading representation in MSTd neurons is affected by depth cues and vestibular translation signals during combinations of real and simulated translation and eye rotation. By varying the virtual environment between a 3D cloud, rich in-depth cues, and a 2D frontoparallel wall devoid of local motion parallax cues and disparity variations, we were able to determine whether depth cues present in the 3D stimulus are required for pursuit compensation. We found some MSTd neurons that are capable of fully or partially compensating for the effects of eye rotation on optic flow without the use of extraretinal signals and without significant differences between the two environments. When vestibular translation cues were added, the amount of compensation was not substantially enhanced. This evidence suggests that pursuit compensation in MSTd depends substantially on visual cues to rotation and does not rely on depth variation to produce local motion parallax cues (see also Yang and Gu, 2017).

Relatively few neurons fully compensated in the simulated rotation condition despite using stimuli rich in dynamic perspective and motion parallax cues. Instead, we see a range of rotation tolerance in MSTd neurons spanning from full compensation to no compensation for simulated and real rotation conditions (Figs. 5, 7). A similarly broad range of rotation tolerance has been observed in previous studies of rotation compensation in MSTd (Yang and Gu, 2017; Manning and Britten, 2019) and VIP (Sunkara et al., 2015). Since the problem that eye rotation poses on the visual system is at least partially solved at the level of human behavior (Warren and Hannon, 1988; Royden et al., 1992), rotation compensation may be solved progressively in the brain at the systems level or, perhaps, complete rotation compensation in visual neurons is not necessary to guide behavior (Cutting et al., 1992). It is also possible that heading estimation is based more strongly on MSTd neurons that show stronger rotation tolerance and that neurons with the weakest rotation tolerance make a lesser contribution to heading perception.

### Behavioral insights to the effects of depth variation on rotation tolerance

Parsing out the heading-informative translational component of optic flow requires eliminating the visual effects of eye rotation. Nonvisual cues to rotation such as proprioception, vestibular inputs, or efference copy of eye, neck, and body movement commands could be used to identify and parse the rotational and translational components of optic flow (Crowell et al., 1998). However, computational models show that heading can theoretically be identified solely from instantaneous optic flow fields that reflect both translation and rotation (for review, see Hildreth and Royden, 1998; Lappe, 2000; Warren, 2008). Such a visual mechanism would eliminate the need to integrate multisensory signals that arrive with varying delays and noise levels (Gellman and Fletcher, 1992; Crowell et al., 1998). Visual models of optic flow analysis often rely on local motion parallax cues between neighboring elements that differ in depth (Longuet-Higgins and Prazdny, 1980; Rieger and Lawton, 1985; Royden, 1997). Since the magnitude of rotational flow vectors is constant across depths, the difference motion vectors formed between pairs of neighboring elements create a radial pattern centered on the direction of heading, even during eye rotation. This retinal strategy requires depth variation; if the visual system relies on this strategy, then compensation for eye rotation should not be possible for the 2D wall environment without extraretinal signals.

These considerations have motivated the use of virtual environments that contain depth variation. Of the behavioral studies that used a 3D cloud of dots, evidence of a purely visual compensatory strategy appears to be sensitive to stimulus parameters and the type of task used to indicate heading. When the ratio of translational to rotational velocities is high, heading judgment errors are typically low but increase with rotational velocity (Warren and Hannon, 1988, 1990; Royden et al., 1992, 1994). The relatively faster rates of rotation in our study are similar to other physiological investigations of rotation compensation (Sunkara et al., 2015; Yang and Gu, 2017) and they were chosen to ensure that changes in tuning curves would be readily measurable for cells that do not compensate for eye rotation. Increasing dot density (Warren and Hannon, 1990) or adding binocular disparity cues (Van den Berg and Brenner, 1994a) improves heading judgements when rotation is visually simulated, which appears to support an important role for motion parallax cues in rotation compensation. Evidence for rotation compensation in studies using a 3D cloud stimulus becomes more prominent as the field of view increases. With visually-simulated rotation conditions, little compensation was found in studies that used a 30° × 30° display (Royden et al., 1994; Banks et al., 1996). With a 40° × 32° display, there was evidence of compensation but only when rotational velocity was quite slow relative to translation (Warren and Hannon, 1988, 1990); with 60° × 50°/55° displays, some evidence of compensation starts to appear under specific conditions (Van den Berg, 1992, 1996; Van den Berg and Brenner, 1994a; Ehrlich et al., 1998).

The 3D clouds used in most psychophysical studies of rotation compensation extend much further in depth than ours by up to 5–40 m (Royden et al., 1994; Banks et al., 1996; Ehrlich et al., 1998). A greater range of depths could be an advantage for mechanisms that compute heading by estimating rotation from the furthest depth planes, which are least affected by translation (Perrone, 1992; Van den Berg, 1992; Van den Berg and Brenner, 1994b). We do not think that the more limited range of depths in our stimuli prevented visual rotation compensation given that some MSTd cells did show near-complete compensation, as did a somewhat greater fraction of VIP cells in a previous study using a similar depth range (Sunkara et al., 2015). According to local motion parallax models, neighboring elements in the foreground are more informative because they contain stronger translational motion components than the background (Longuet-Higgins and Prazdny, 1980; Warren, 1998). The dot density and depth of our 3D cloud provided these cues and are broadly similar to other physiological studies (Sunkara et al., 2015; Yang and Gu, 2017; Manning and Britten, 2019).

Of the few behavioral studies that used a 2D frontoparallel wall stimulus, large heading errors resulted from stimulus displays that subtended ≤45° of visual angle during simulated pursuit, but not during real pursuit (Rieger and Toet, 1985; Warren and Hannon, 1988, 1990; Royden et al., 1992, 1994; Grigo and Lappe, 1999). However, Grigo and Lappe (1999) used a 90° × 90° display with a 2D frontoparallel wall stimulus and found very small heading biases during simulated rotation for short stimulus durations. This finding supports a visual mechanism of rotation tolerant heading estimation that does not rely on local motion parallax cues. Simulated rotation produces a deformation of the flow field under planar projection that can potentially be used to dissociate translation and rotation (Koenderink and Van Doorn, 1975, 1976, 1981). These rotational cues, which have also been referred to as dynamic perspective (Kim et al., 2015), are stronger in the periphery which may explain why a large field of view results in stronger rotation compensation (Koenderink and van Doorn, 1987; Grigo and Lappe, 1999). Since local motion parallax cues should be effective even in smaller displays, dynamic perspective cues might have been the driving influence behind rotation compensation effects that grew with display size in studies using 3D cloud stimuli, as described above. Unfortunately, behavioral studies that used 3D clouds did not have display sizes that exceeded 60°, so the evidence remains somewhat equivocal. Importantly, however, the idea that dynamic perspective cues, rather than local motion parallax cues, may be critical for rotation compensation is compatible with our finding that rotation tolerance of heading tuning in MSTd was not enhanced in the 3D cloud environment.

### Previous electrophysiological evidence of a visual compensation strategy

Only a couple of previous studies have compared the effects of 3D and 2D visual environments on rotation tolerance of heading tuning, and they both had notable limitations. Sunkara et al. (2015) investigated pursuit compensation in VIP neurons using stimuli similar to our 2D and 3D environments, but this was done in separate experiments on different sets of neurons. They found significantly greater compensation in the 3D environment but both environments resulted in subpopulations of neurons that fully compensated and some that partially compensated. This shows that retinal information is sufficient for rotation tolerant heading responses in a subpopulation of VIP neurons. However, since the comparison was made between separate populations of neurons in VIP, it remains uncertain that the greater compensation seen for the 3D cloud environment implies a specific role of motion parallax cues. While the finding of Sunkara et al. (2015) suggests a sensitivity to motion parallax cues in VIP that we did not find in MSTd, the effect might have arisen from different sampling in the two populations they studied.

Yang and Gu (2017) measured pursuit compensation in MSTd during real rotation only, varying the presence and absence of motion parallax cues in separate blocks of trials. Using a very similar analysis of tuning curve shifts as Sunkara et al. (2015), Yang and Gu found that motion parallax cues in their 3D cloud environment slightly enhanced rotation compensation in MSTd neurons, although the effect was just shy of statistical significance. However, since eye rotation was always real pursuit in the experiment of Yang and Gu, and since this non-visual input apparently drove substantial compensation, they speculated that motion parallax cues might have a greater impact when rotation is visually simulated. In our experiments, motion parallax cues in the 3D cloud condition did not enhance rotation tolerance for either real or simulated rotations, suggesting that motion parallax cues play little role in creating rotation tolerant heading tuning in area MSTd.

Instead of dissociating visual and extraretinal signals by using simulated rotation, Manning and Britten (2019) inverted the cue conflict by eliminating the rotational component of optic flow during eye rotation. In this stabilized pursuit condition, an extraretinal rotation signal is accompanied by a visual signal that lacks rotation cues. Their real and simulated rotation conditions, both of which presented nearly the same visual rotation cues, resulted in partial compensation with modestly larger shifts in tuning for simulated rotation. However, the stabilized pursuit condition resulted in no significant shifts of the tuning curves despite the presence of an extraretinal rotation signal. This study provides additional evidence that the visual rotation signal is the dominant component in the neural compensatory mechanism in MSTd. Since Manning and Britten did not repeat their experiments using a 2D frontoparallel plane stimulus that lacks local motion parallax cues, we cannot tell whether the visual mechanisms rely on motion parallax or dynamic perspective cues that were also ample in their large display (100° × 68°).

Combined with our results, it is clear that visual motion independently supports rotation tolerant heading representation in some MSTd neurons, and that dynamic perspective cues are sufficient indicators of eye rotation, as has also been shown for computation of depth sign in area MT (Kim et al., 2015).

### Vestibular contributions to heading mechanisms during eye rotation

The addition of vestibular heading cues to optic flow led to a modest, and marginally significant, enhancement of pursuit compensation for the subset of MSTd neurons with vestibular heading tuning, but did not have an effect at the level of the entire population. This was somewhat surprising given the presence of vestibular heading signals in MSTd (Duffy, 1998; Bremmer et al., 1999; Gu et al., 2006) and the increase in sensitivity for heading discrimination in humans (Butler et al., 2015; Crane, 2017) and monkeys (Gu et al., 2008; Fetsch et al., 2009) when congruent vestibular cues are added to translational optic flow. Vestibular signals also contribute to the dissociation of object motion from self-motion at both the perceptual (Fajen and Matthis, 2013; Dokka et al., 2015a,b, 2019) and neuronal (Sasaki et al., 2017, 2020) levels. Since the effect of vestibular translation signals on rotation compensation has not been studied in other areas, we cannot rule out the possibility that vestibular cues contribute more substantially to rotation invariant heading tuning in downstream areas such as VIP, which also receives vestibular inputs (Bremmer et al., 2002; Chen et al., 2011).

To our knowledge, this is the first investigation of the contribution of vestibular translation signals to the rotation tolerance of heading tuning. However, a few studies have investigated the role of vestibular rotation signals in heading judgements made during head rotations. Crowell et al. (1998) used a heading discrimination task to measure rotation compensation in humans during simulated translation in a constant direction plus various combinations of simulated and real (active and passive) head rotations designed to isolate combinations of visual, vestibular, proprioceptive, and efference copy signals. The added vestibular rotation cues were the least effective extraretinal signal, resulting in a mere 4% increase in compensation compared with optic flow alone. Compensation was maximized at a 94% increase when all three extraretinal cues to rotation were available. This shows that when adequate visual cues to rotation are not available, extraretinal cues to rotation help to reduce the effects of rotation on heading perception, but vestibular rotation cues alone are not sufficient.

At the neural level, Shenoy et al. (1999) measured the stability of heading tuning curves in MSTd during simulated translation combined with passive, full body rotation while canceling the vestibulo-ocular reflex (VORC condition). This condition adds vestibular rotation cues to rotational optic flow while the eyes are fixed in the head. The amount of rotation compensation in the VORC condition (77.2%) did not differ significantly from the real pursuit condition (88.4%), for which the extraretinal signal comes from rotation of the eye in the head without vestibular cues. Both conditions resulted in significantly greater compensation than during simulated pursuit when only optic flow was available (52.0%). The weaker compensation observed during simulated rotation was likely affected by the use of a small stimulus display (18° × 18°) as well as the fact that laminar motion was used (incorrectly) to simulate eye rotation, thereby failing to provide the dynamic perspective cues that should generally accompany eye rotation (see Sunkara et al., 2015).

The vestibular rotation signals in these previous studies can help to estimate the rotational component of self-motion. Our study differs in that we have provided translational vestibular signals that could be used to directly estimate heading when optic flow is altered by pursuit eye movements. The vestibular translation cues did not substantially improve the amount of compensation achieved from purely visual inputs to MSTd, although there were small effects for neurons with stronger vestibular heading tuning. This result is unlikely to reflect a ceiling effect in the amount of compensation achievable at this level of the visual system, given that most MSTd neurons in our study were not close to showing full compensation for rotation. Thus, our finding may suggest that vestibular translation signals have a greater influence on rotation compensation at some other stage of processing, or that they simply do not make a major contribution to this process.

### The relationship between rotation selectivity and rotation compensation

Compensating for eye rotation during translation involves canceling the effects of rotational optic flow to represent the heading-informative translational component. Accordingly, our analysis of rotation selectivity in MSTd showed that neurons with strong rotation compensation were less selective to pure rotation under the same visual rotation conditions (Fig. 8*B*). Likewise, neurons with weak rotation compensation during translation were more likely to be selective to pure visual rotation stimuli.

There are two basic ways that one can conceptualize this finding. One is that responses of rotation-tolerant MSTd neurons undergo a transformation that reduces their sensitivity to rotational optic flow, perhaps via signals from other neurons or areas that actively suppress some inputs to these neurons. In this scenario, suppression of the rotational flow sensitivity would carry over to the pure rotational control conditions, thus leading to small DDI values in the pure rotation control conditions for rotation-tolerant neurons (Fig. 8*A*,*B*). A second possible way to conceptualize this finding is that rotation-tolerant MSTd neurons generally lack excitatory inputs that are sensitive to rotational optic flow. In this case, the correlation between rotation compensation (tuning shift) and DDI (Fig. 8*A*,*B*) would arise because tolerant neurons lack bottom-up inputs sensitive to pure visual rotation, not because of some kind of suppression. While we cannot rule out either possibility, we tend to favor the former explanation because the rotational component of optic flow (Fig. 1*A*) contains a strong laminar motion component that should tend to strongly activate inputs to MSTd from area MT, and thus may need to be actively suppressed somehow to generate rotation-tolerant heading tuning.

If MSTd neurons relied mainly on extraretinal rotation signals to identify the rotational component of optic flow, we might expect neurons with rotation-tolerant heading tuning during real eye rotation to have reduced selectivity to pure eye rotation in darkness. On the contrary, we found no significant relationship between compensation and rotation selectivity in darkness (Fig. 8*C*), which also suggests that rotation compensation may not rely heavily on extraretinal rotation signals.

### MSTd population estimates of heading during eye rotation

We used an OLE to decode heading from MSTd population activity and compared estimation biases between the two virtual environments (2D, 3D) and the two translation conditions (visual, visual with vestibular). Our methods were motivated by the likely constraint that a single set of decoding weights is optimized to estimate heading in the absence of rotation, and that the same weights are applied to estimate heading when eye rotations occur. The decoding results for 3D versus 2D environments and real versus simulated translation were quite consistent with the conclusions derived from our single cell analyses. Although we observe a large range of rotation tolerance across the population of single neurons, our decoding results provide no clear evidence that a particular strategy of rotation compensation benefited from selectively weighting responses from a subset of MSTd neurons.

Ben Hamed et al. (2003) also used an OLE to decode heading from population activity in MSTd during simulated translation and real eye rotation, and they report an average error of <2° on rotation trials. The OLE in their study was trained on 10,000 bootstrapped responses from 144 neurons and tested on bootstrapped responses sampled from two withheld repetitions of the same conditions as the training set. By comparison, our OLE was trained on the trial-averaged responses of 58–60 MSTd neurons to pure translation and was tested on bootstrapped responses to combined translation and simulated rotation. The greater accuracy found by Ben Hamed et al. (2003) is likely because of the fact that they trained and tested their decoder on responses to the same set of conditions containing both visual and extraretinal cues to rotation. Thus, our decoding analysis tests generalization to the conditions with rotation, whereas theirs did not.

In conclusion, evidence favoring a visual strategy of translational and rotational optic flow decomposition has been accumulating in the literature, suggesting that visual mechanisms may dominate the process for estimating heading in the presence of eye rotations (Lappe et al., 1999; Crowell and Andersen, 2001; Wilkie and Wann, 2002; Manning and Britten, 2019). However, it has remained unclear which visual cues the visual system relies on to construct rotation-tolerant heading tuning. Our results suggest that dynamic perspective cues available in both the 2D and 3D environments may be the critical visual cue to eye rotation, and that the addition of local motion parallax and disparity cues within the 3D environment does not improve rotation compensation in MSTd. Our results also suggest that vestibular cues to translation do not make a major contribution to the compensatory mechanism in MSTd. These findings further support a visual strategy capable of at least partially compensating for the effect of eye rotation in heading estimation.

## Synthesis

Reviewing Editor: Satu Palva, University of Helsinki Decisions are customarily a result of the Reviewing Editor and the peer reviewers coming together and discussing their recommendations until a consensus is reached. When revisions are invited, a fact-based synthesis statement explaining their decision and outlining what is needed to prepare a revision will be listed below. The following reviewer(s) agreed to reveal their identity: NONE.

abstract

This is a nice and exhaustive study on the role of MSTd in estimating heading direction in the presence of body translation combined with eye ball rotation. The authors show evidence supporting visual mechanisms for compensation of eye rotation when inferring heading direction. They do this by collecting full tuning curves with and without real eye movements and under different 2d/3d viewing conditions. They also rely on population decoding approaches. The experiments appear to show that many cells can compensate for real eye movements in the absence of depth cues (i.e. during heading toward a 2D Wall) and a few cells can compensate for simulated eye rotations in the absence of depth cues. Adding depth cues, in the form of a 3D cloud of dots did not increase the compensation for rotation in either real or simulated conditions. Adding real vestibular cues, using a moving platform, also did not increase compensation for eye rotation. This is an interesting result, particularly since it challenges the predictions of many models of heading perception that rely on depth variation in the scene to accurately compute translational heading in the presence of eye rotations. It is particularly interesting since it also seems counter to psychophysical results that suggest that depth aids humans in compensating for simulated eye rotations when judging heading. It may be that this result is limited to the conditions used here, i.e. a very large field of view, binocular disparity information available in the display, a narrow range of depths in the 3D cloud, and rather fast rotation rates. The experiments appear to be well thought out and the analysis seems appropriate. There are a few things that the authors should attend to before publication of this manuscript.

Abstract and significance statement.

Abstract suggest that visual cues (but not necessarily local motion parallax) account for the rotation tolerance. The significance statement now says that “visual depth cues” have little effect on rotation tolerance, which is quite confusing. It might be best to slightly modify the wording for clarity, especially since the reader has typically not read the rest of the paper at this stage.

1) As I noted, the results in this paper may depend on the specific conditions used. As the authors note, the compensation for simulated eye rotation in the absence of depth variation requires a large field of view so that the differences between the translation + rotation stimulus and a translation stimulus with a shifted heading can be detected. Smaller fields of view will likely lead to different results, at least with respect to cells being able to compensate for eye rotations in the simulated rotation condition. Even with the large field of view, very few cells fully compensated for the rotations in the simulated rotation condition. The authors should state this limitation of the study clearly.

2) The stimuli with added depth have a rather small range of depths compared to stimuli used in psychophysical studies, with the range only between 10 and 80 cm. It is possible that this range of depth is too small to have an appreciable effect on the rotation compensation, particularly since the tuning curves are so broad that a small effect might not be observable or rise to the level of statistical significance. The authors should note this in their discussion.

3) The stimuli have binocular disparity cues and very high rates of rotation, (15 or 22 deg/sec which are higher than rotation rates used in many psychophysical studies). The disparity cues and high rates of rotation also may be a required for the compensation they are seeing. They should note this as well in the discussion. It would be interesting, in future studies, to examine whether compensation can occur in the absence of disparity cues or with slower rotation rates.

4) The authors refer to “dynamic perspective,” referring to distortions in the optic flow field that can be used to determine the rate of eye rotation. Because this concept is so important to understanding the results, it would be useful for the authors to describe it in some more detail, rather than simply citing papers. A paragraph or two, and perhaps a figure, describing this cue and how it can be used to identify rotation rates would be very helpful to readers of this paper, and make the manuscript more self-contained.

5) p. 8, lines 176-177: The authors refer to the pursuit target as projected onto the display at zero binocular disparity. What is the simulated fixation distance? I.e. where is zero disparity with respect to the depths of the dots on the screen? What is the range of disparities for each of the stimuli used in the experiments (at the start and end of each trial)?

6) p. 11, lines 257- 260: The sentence on these lines is not clear. What do the authors mean when they say “simulated translation in the 2D wall environment was nested with real and simulated eye rotation conditions...”? This sentence needs to be re-written.

7) In the analysis section, the authors start referring to “curve half” without defining what they mean by it. It took me a while to figure out what part of the curve they were referring to as the “curve half.” They should define it clearly when they first use the term.

8) p. 14, lines 355-357: The authors state that “extensive visual inspection was used” to confirm that the shift values were reliable. What were the criteria they used for this visual inspection? This seems rather subjective, and could result in biased conclusions.

9) p. 18, line 445: How did the authors convert polar coordinates to 2D Cartesian coordinates for the 90 degree or 270 deg headings, for which the heading goes to infinity?

10) Figures 4 and 6: It would be helpful to separate out the data for the real rotation and the simulated rotation into separate graphs. It’s difficult to see many of the points, as there are so many clustered together, and putting them on separate graphs may allow readers to see similarities and differences between the two conditions more readily.

11) Figure 7 (and related text): The authors refer to a condition for the DDI measurement of “real rotation with optic flow”. Perhaps I missed this in the methods section, but on page 28, line 667 refers to rotation with a blank background or a visual background of dots. It doesn’t say that the dots are moving, so I assumed they were stationary (on the screen). If they are moving (e.g. with a simulated translation), the authors should state that clearly. If the optic flow is generated only from the rotation, they should make that clear.

12) page 40, lines 948-949: The authors refer to “an incorrect method for visually simulating rotations,” and giving a citation. It would be useful to describe briefly here what the method was and why it was incorrect.

13) Introduction first refers to Fig. 1A, D before the methods section later describes panels B and C. It might make sense to re-organize the figure slightly such that panel D is actually panel B, and then panels B and C become panels C and D. This way, the reference to the figure panels in the text comes in order. This would also help reading the figure itself since the figure is now kind of organized in a non-conventional order (top to bottom and then to the right along the bottom and then back to top when cycling through all of the individual panels A to D).

14) line 164: it would help to be a bit more explicit here. e.g. was the entire scleral search coil cube and display moved with the chair?

15) line 228: I was lost here at “5 rotation conditions”. I guess there were so many condition descriptions in the previous two paragraphs, that I got lost here. I can understand that 2 rotation directions are clear, but I was lost what are the 5 rotation conditions? 2d/3d and real/simulated gives 4 conditions, no?

16) line 277: I wonder why only 200 Hz for digitizing the eye signals?

17) line 296: should “turning” be “tuning”?

18) Fig. 2C: I had a bit of a hard time deciphering whether this change is indicative of visual mechanisms or extra retinal compensation? This became clearer later at the beginning of the Results section. So, maybe a reference to “see Results” or something like that is sufficient here.

19) In general, the text right after Fig. 2 got a bit complex. A figure with an example neuron’s tuning curves and panels explaining the analysis steps would be very useful here.

20) line 557: There are multiple related terms that are used interchangeably by the authors, but that can cause strong confusion. For example, the abstract started with the idea of “tolerance", then methods abandoned this term in favor of others like “shifts” or “compensation", and now in Results, “compensation”. This is quite confusing to the readers, and it shows a disconnect between different sections of the paper.

21) Fig. 4: does the fact that shifts were less than the predicted value of ˜26–28 deg mean that there is some partial compensation extra-retinally?

22) Can the authors mention how they addressed saccades in terms of the analyses? Also, more generally, maybe they can comment on how the neurons will represent heading direction during saccades (fast rotations), if at all.

23) it would be nice if the paper showed some raw eye movements and neural responses before the first tuning curves.
